# Comprehensive mapping of somatotroph pituitary neuroendocrine tumour heterogeneity using spatial and single‐cell transcriptomics

**DOI:** 10.1002/ctm2.70090

**Published:** 2024-11-15

**Authors:** Jialin Wang, Xuejing Li, Jing Guo, Zan Yuan, Xinyu Tong, Zehao Xiao, Meng Liu, Changxiaofeng Liu, Hongyun Wang, Lei Gong, Chuzhong Li, Yazhuo Zhang, Weiyan Xie, Chunhui Liu

**Affiliations:** ^1^ Department of Cell Biology, Beijing Neurosurgical Institute Capital Medical University Beijing China; ^2^ Annoroad Gene Technology (Beijing) Co., Ltd Beijing China; ^3^ Department of Neurosurgery Beijing Tiantan Hospital Affiliated to Capital Medical University Beijing China

**Keywords:** somatotroph PitNETs, spatial transcriptomics, single‐cell RNA sequencing, tumour heterogeneity

## Abstract

**Background:**

Pituitary neuroendocrine tumours (PitNETs) are common intracranial tumours that are highly heterogeneous with unpredictable growth patterns. The driver genes and mechanisms that are crucial for tumour progression in somatotroph PitNETs are poorly understood.

**Methods:**

In this study, we performed integrative spatial transcriptomics (ST) and single‐cell RNA sequencing (scRNA‐seq) analysis on somatotroph tumours and normal pituitary samples to comprehensively characterize the differences in cellular characteristics.

**Results:**

By analyzing combined copy number variations (CNVs), tumour tissues were divided into two regions, which included the CNV_high_ and CNV_low_ areas. The protumour genes DLK1 and RCN1 were highly expressed in the CNV_high_ area, which might be related to tumour progression and could be targeted for precision therapy. We also found that the transforming growth factor beta signalling pathway participated in tumour progression and identified heterogeneity in the expression profiles of key genes. We assessed the intertumoral and intratumoral heterogeneity in somatotroph PitNETs and emphasized the importance of individualized treatment.

**Conclusion:**

In summary, we visualized the cellular distribution and transcriptional differences in normal pituitary and somatotroph PitNETs by ST and scRNA‐seq for the first time. This study provides a strong theoretical foundation to comprehensively understand the crucial mechanisms involved in tumour progression and develop new strategies to treat somatotroph PitNETs.

**Key points:**

- The first‐ever visualization of cellular distributions in normal and tumor pituitary tissues.
- The inter‐ and intra‐tumoral transcriptomic heterogeneity of somatotroph PitNETs was comprehensively revealed.
- Identification of potential protumor factors and critical signaling pathways, opening new avenues for therapeutic intervention.

## 1 INTRODUCTION

Pituitary neuroendocrine tumours (PitNETs) are common and complex intracranial tumours with high heterogeneity. The prevalence of PitNETs ranges from 80 to 100/100,000 people to 1/1000 people, of which 9%–14% are somatotroph PitNETs/growth hormone (GH)‐producing pituitary adenomas.^1^, ^2^ Acromegaly, caused by somatotroph PitNETs, is characterized by overgrowth in certain parts of the body, such as the tongue, distal extremities and facial changes. Severe cases are accompanied by complications, such as cardiomyopathy, osteoarthritis, diabetes, hypertension and nerve entrapment syndromes, which may lead to high mortality.^3^, ^4^ Around 50% of acromegaly patients cannot achieve surgical cure and require adjuvant therapy.^5^ In one‐third of patients treated with somatostatin analogues, acromegaly cannot be brought under control.^6^ Even after undergoing surgical resection and medical therapy, achieving remission in these patients is difficult, probably because somatotroph PitNETs are heterogeneous.^4^, ^7^ Therefore, a comprehensive analysis of the cellular composition and spatial heterogeneity of somatotroph PitNETs may help identify effective targets and elucidate the mechanism of tumour progression.

Single‐cell RNA sequencing (scRNA‐seq) is an increasingly popular technique to characterize PitNETs and various solid tumours.^8^, ^9^, ^10^, ^11^, ^12^ Recent advancements in scRNA‐seq have allowed researchers to investigate intratumoral heterogeneity in pituitary tumours. For example, our study confirmed the intrinsic cellular heterogeneity of the tumour cells and the tumour microenvironment (TME).^13^ Single‐cell multi‐omics sequencing approaches have shown the heterogeneity in copy number variations (CNVs) in PitNETs; thus, revealing significant intratumoral genomic heterogeneity.^14^ Additionally, in a study, the functional and cellular heterogeneity of immune cells and tumour‐associated fibroblasts (TAFs) in PIT1‐positive pituitary adenoma was investigated by scRNA‐seq.^15^ However, scRNA‐seq alone cannot provide the spatial information needed to correlate transcriptional state dynamics with tumour topography.^16^ Spatial transcriptomics (ST) is a transformative technology that reveals complex spatial structures with transcriptome data and spatial information in tumours.^17^, ^18^, ^19^, ^20^, ^21^ ST has been used successfully to study tumours, such as oral squamous cell carcinoma, liver cancer, melanoma and prostate cancer.^9^, ^16^, ^22^, ^23^ ST provides key information to understand tumour heterogeneity, identify new biomarkers, optimize treatment strategies and decipher the dynamic TME.^24^ However, the intratumoral spatial heterogeneity in PitNETs remains poorly understood. Additionally, PitNETs have different types of cells and growth patterns characterized by expansion into or infiltration of surrounding parasellar tissues. Although PitNETs, including sparsely granulated somatotroph PitNETs, are mostly benign, they may exhibit aggressive clinical behaviour, resulting in high recurrence rates and resistance to standard therapy, suggesting the complexity of PitNETs.^25^ Thus, the heterogeneity and spatial cell distribution of PitNETs need to be investigated using ST.

In this study, we have used ST combined with scRNA‐seq to examine intratumoral transcriptional heterogeneity within somatotroph PitNETs by characterizing and identifying their unique geographic structures. Our findings indicated that somatotroph tumours exhibit higher spatial heterogeneous expression of angiogenesis‐related and tumour invasion‐related genes compared to normal pituitary tissues. Additionally, CNVs in tumour tissues exhibited spatial heterogeneity. Analyzing CNVs can help distinguish between tumour and paratumour areas. By conducting a differential analysis based on the transcriptional expression patterns of the CNV_high_ and CNV_low_ regions, we further identified crucial genes related to tumour progression, such as DLK1 and RCN1. Combined with the scRNA‐seq data, we found spatial heterogeneity in the transforming growth factor beta (TGF‐β) signalling pathway. We also observed that the tumour tissue was in a state of immune infiltration, unlike the normal pituitary tissues. Moreover, the tumour region was immunosuppressed, unlike the paratumoural region, at the spatial level. In this study, ST provided new insights into the immune environment of somatotroph PitNETs. To summarize, we provided spatial information on somatotroph PitNETs for the first time and decoded the complex somatotroph PitNET landscape.

## 2 RESULTS

### 2.1 Overview of spatial and single‐cell transcriptome analysis of somatotroph PitNETs

**FIGURE 1:**
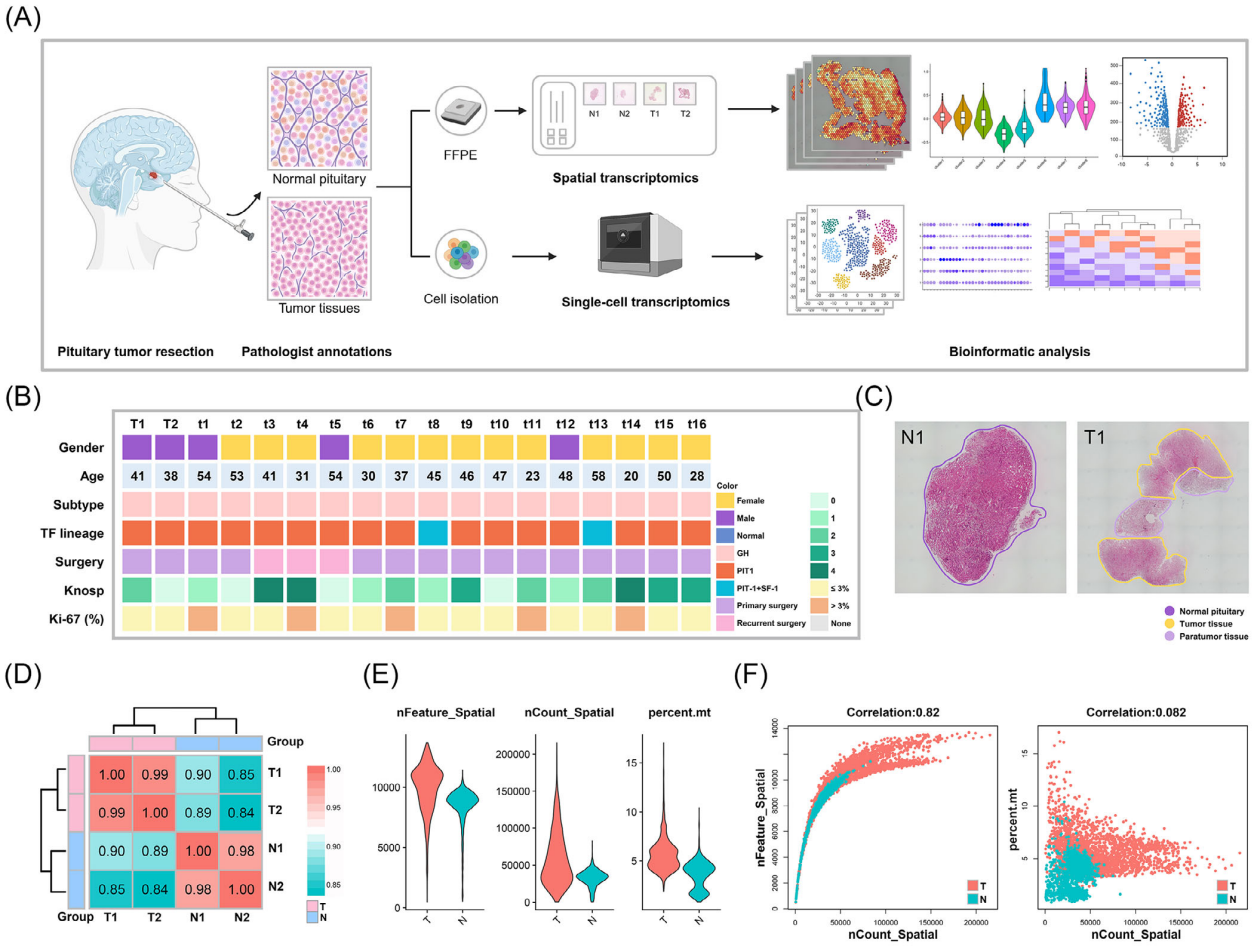
Overview of the study design and information on spatial transcriptomics (ST) and single‐cell RNA‐sequence (scRNA‐seq). (A) Schematic overview of the study design. After the pathologist identified samples, normal pituitary and tumour tissues were processed with 10x Genomics Visium to obtain transcriptomics data for the subsequent analytical workflow. The image was created in BioRender.com. (B) Clinical information of the patients. PIT‐1+SF‐1: both PIT‐1 and SF‐1 were positive tumours. (C) H&E‐stained images of N1 and T1 samples annotated by pathologists. The T1 sample contained the tumour area and the adjacent paratumour area. (D) Correlation analysis of spatial transcriptome samples. (E) The violin plots show the quality control of spatial transcriptomics data. (F) Spatial spots were strongly correlated with genes, but they were not correlated with mitochondrial genes. N, normal pituitary; T, tumour tissue; TF, transcription factor.

Spatial information can help understand tumour progression. However, such information on PitNETs is absent. To obtain the spatial distribution landscape of somatotroph PitNETs, we have collected four formalin‐fixed paraffin‐embedded (FFPE) tissue samples, including two somatotroph PitNETs (T1 and T2) and two normal pituitary tissues (N1 and N2), from four patients for 10X Genomics Visium spatial transcriptome analysis. Additionally, we selected 20 scRNA‐seq data from 20 patients for auxiliary validation analysis, including 16 somatotroph PitNETs (t1–t16) and four normal samples (n1–n4) (Figure 1A and Table S1). The clinicopathological information of these patients is summarized in Figure 1B. The tumour tissue, paratumour tissue and normal pituitary tissue were identified by hematoxylin and eosin (H&E) staining and annotated by pathologists (Figure 1C and Figure S1). Data from the unsupervised clustering similarity matrix was used to confirm consistency with pathological identification (Figure 1D). In total, 4449 spots were sequenced from four samples. After strict quality control, 4,426 high‐quality spots were further analyzed (Figure 1E). A strong correlation was found between spatial spots and genes (correlation = 0.82), while no correlation was observed between spatial spots and mitochondrial genes (correlation = 0.082) (Figure 1F).

### 2.2 Spatial annotation and distribution profile of somatotroph PitNETs and normal pituitary tissues

**FIGURE 2:**
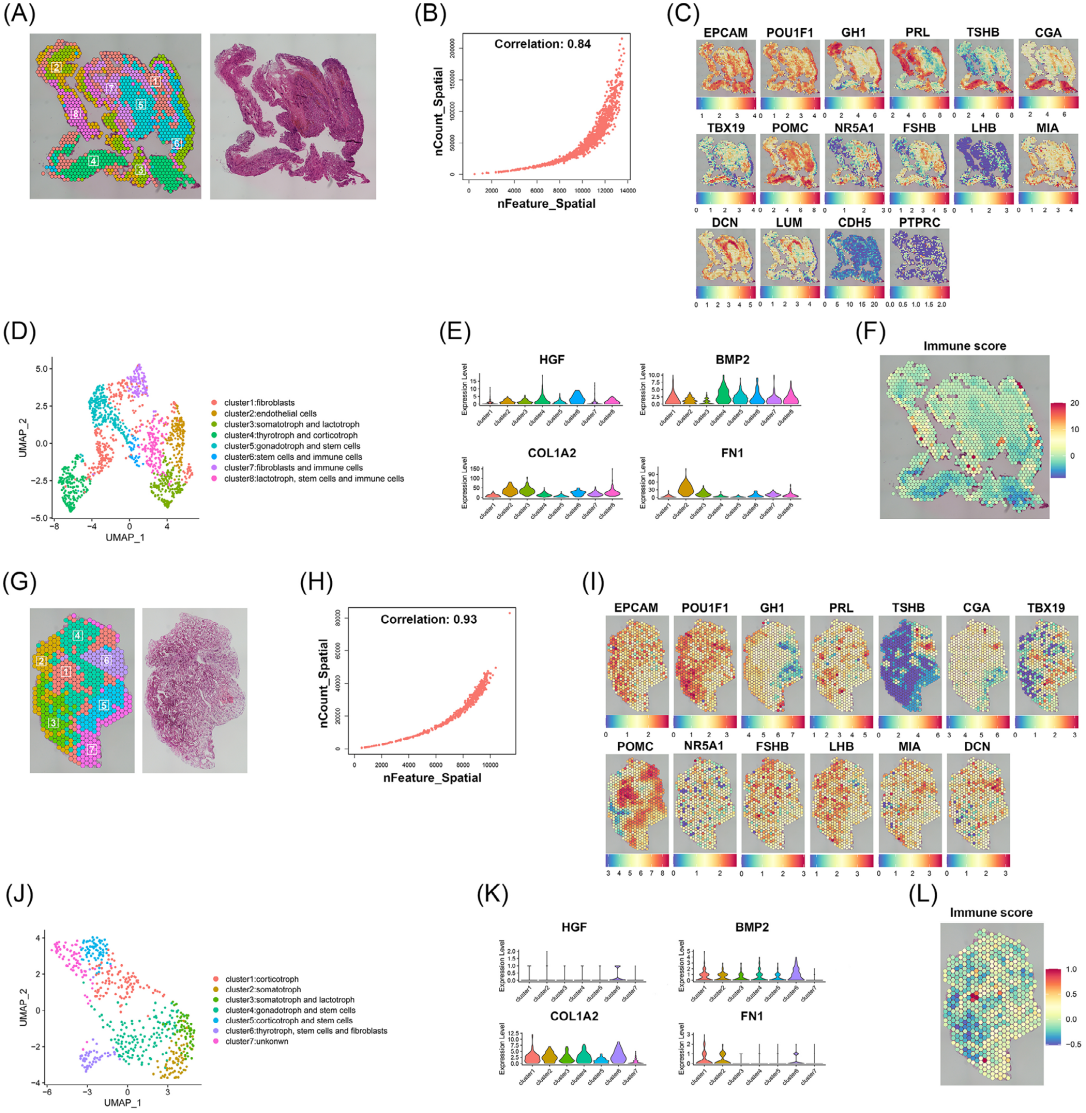
Identification and characterization of cells in normal pituitary samples and somatotroph tumours. (A–F) show results related to the tumour tissue (T2). (A) Spatial transcriptomic section map of T2 sample. Unbiased clustering of spatial transcriptomics (ST) spots and H&E staining. (B) The scatter plot illustrates the correlation between spatial spots and genes. (C) Spatial plots of the known markers for somatotroph, lactotroph, thyrotroph, corticotroph, gonadotroph, stem cells, fibroblasts, endothelial cells and immune cells. (D) UMAP plot of the cellular landscape of the T2 sample. (E) The violin plots show the expression of genes related to angiogenesis and tumour invasiveness (HGF, BMP2, COL1A2 and FN1) in clusters. (F) The violin plot shows the immune scores across the clusters. (G–L) Results related to the normal pituitary (N2), using the same representation as tumour tissue (T2). (G) Spatial transcriptomic section map of N2 sample. (H) Scatter plot demonstrates the correlation between the spatial spots and genes of the N2 sample. (I) The spatial distribution of the known markers for somatotroph, lactotroph, thyrotroph, corticotroph, gonadotroph, stem cells and fibroblasts. (J) The UMAP plot of the cellular landscape of the N2 sample. (K) The violin plots illustrate the expression of genes related to angiogenesis and tumour invasiveness (HGF, BMP2, COL1A2 and FN1) in clusters. (L) The violin plot shows the distribution of the immune scores across the clusters of the N2 sample. N, normal pituitary; T, tumour tissue.

To comprehensively assess the cellular characteristics of different regions, images of H&E‐stained sections with distinct histological features were observed, and unsupervised clustering was performed at the spatial level. The first ST map of the somatotroph PitNET tissue (T2) is shown in Figure 2A. Genes and spatial spots were strongly correlated (Figure 2B). Using marker‐based annotations, epithelial cells (EPCAM), somatotroph (POU1F1 and GH1), lactotroph (POU1F1 and PRL), thyrotroph (POU1F1, TSHB and CGA), corticotroph (TBX19 and POMC), gonadotroph (NR5A1, FSHB, LHB and CGA), stem cells (MIA), fibroblasts (DCN and LUM), immune cells (PTPRC) and endothelial cells (CDH5) were identified (Figures 2C and Figure S2).^8^, ^13^, ^14^, ^15^, ^26^ This information was used to describe the overall cellular composition of somatotroph tumours (Figure 2D). We found that the levels of protumour factors involved in tumour invasiveness and angiogenesis, such as HGF,^27^, ^28^ BMP2,^29^, ^30^ COL1A2^31^ and FN1,^32^ were higher across cell types in the T2 tumour sample compared to their corresponding levels in normal samples (Figure 2E). We also evaluated the immune scores, which indicated immune cell infiltration in somatotroph PitNETs (Figure 2F). This finding provided visual evidence for the presence of immune infiltration in PitNETs. The first ST map and H&E‐stained image of the normal pituitary (N2) is shown in Figure 2G. The correlation coefficient for the correlation between genes and spatial spots was 0.93 (Figure 2H). Markers for epithelial cells, somatotroph, lactotroph, thyrotroph, corticotroph, gonadotroph, stem cells and fibroblasts are shown in Figures 2I,J and Figure S2. However, low levels of expression of genes related to tumour invasiveness and angiogenesis (Figure 2K) and low immune scores (Figure 2L) were noted in the normal pituitary (N2). Similar results were obtained for two additional samples (N1 and T1) (Figure S3). Furthermore, immunohistochemistry was performed on an immune cell marker (PTPRC/CD45), which found that the tumour region is immunosuppressed compared to the paratumoural region. However, there is an infiltration of immune cells in the tumour tissue compared to the normal tissue (Figure S8E,F). This was the first study in which the cellular diversity and immune status of somatotroph PitNETs and normal pituitaries were visualized at the spatial level.

### 2.3 Spatial characterization of the differential gene expression profile in somatotroph PitNETs and normal pituitary

**FIGURE 3:**
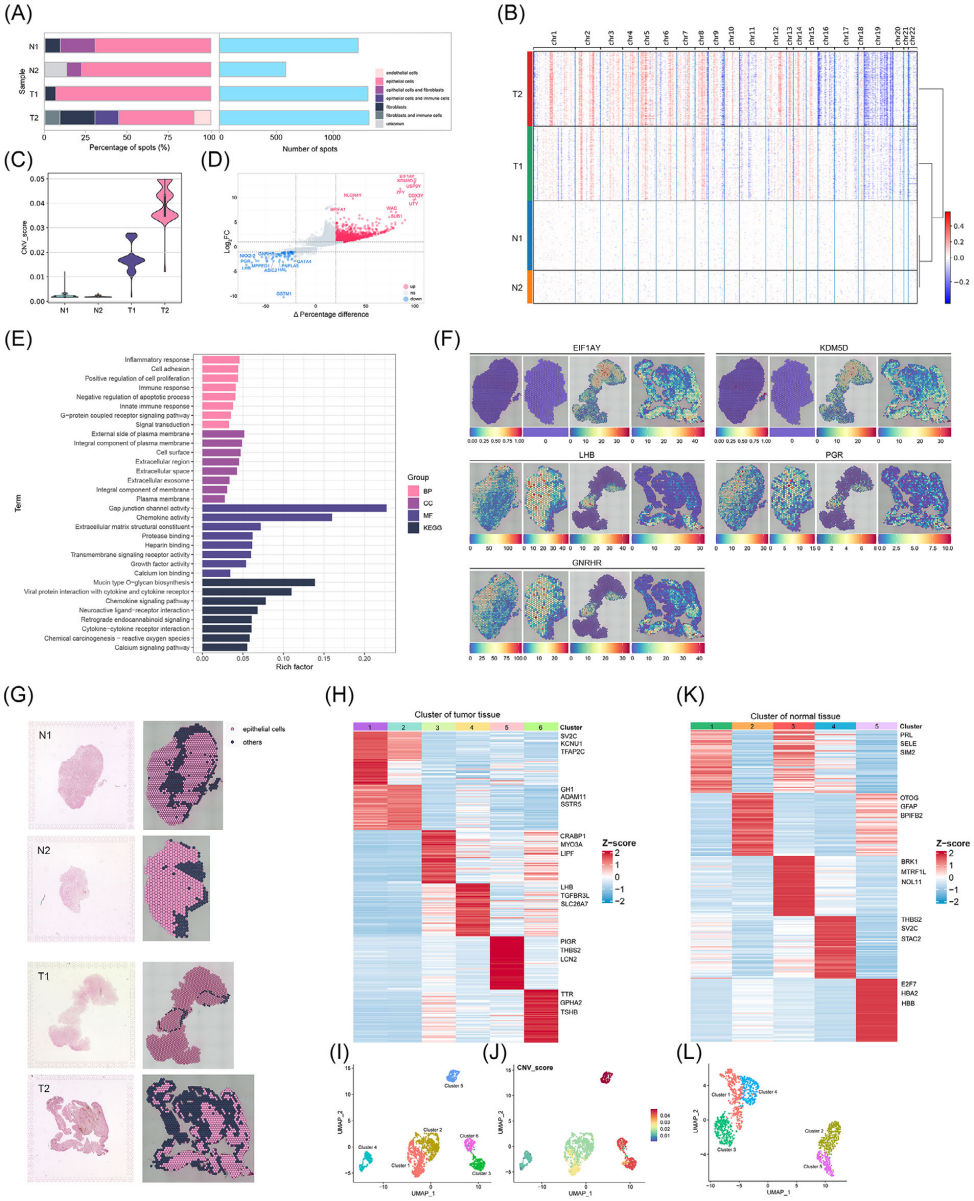
Differential characteristics in normal pituitary and somatotroph tumours based on spatial transcriptomics. (A) The bar plots show the percentage of four major cell types (left) and the number of spatial spots (right) in each sample. (B) A heatmap of the copy number variation (CNV) profile inferred from spatial transcriptomics in spatial spots from each sample. (C) The violin plot shows the CNV scores of each sample. (D) The volcano plot shows differentially expressed genes (DEGs) between the tumour tissue and the normal pituitary. (E) The bar plot represents the Gene Ontology (GO) and Kyoto Encyclopedia of Genes and Genomes (KEGG) enrichment analysis of upregulated DEGs of tumour tissue. (F) The spatial distribution of EIF1AY, KDM5D, LHB, PGR and GNRHR in four samples was determined by ST. (G) H&E staining of ST sections (left). Epithelial cell distributions in four samples (right). (H) The heatmap shows cluster‐specific genes in tumour tissue. (I) The UMAP plot depicts the unbiased clustering of tumour tissue. (J) UAMP plot shows the CNV levels in tumour tissue. (K) The heatmap illustrates cluster‐specific genes in the normal pituitary. (L) The UMAP plot shows unbiased clustering in the normal pituitary.

Next, we examined the differences in the characteristics between somatotroph tumours and the normal pituitary. The cells were divided into seven major cell types, including endothelial cells (*n* = 133), epithelial cells (*n* = 3127), fibroblasts (*n* = 475), epithelial cells and fibroblasts (*n* = 309), epithelial cells and immune cells (*n* = 188) and fibroblasts and immune cells (*n* = 118) and unknown cell type (*n* = 76) (Figure 3A). The epithelial cells were the predominant cell type in all samples. However, neither immune cells nor endothelial cells were identified in normal pituitary samples, which suggested that changes might have occurred in the tumour microenvironment of PitNETs. Genomic CNVs and CNVs inferred from scRNA‐seq have been reported in PitNETs, which are not analyzed in the spatial transcriptome data. We extrapolated CNVs from spatial transcriptome data and found that the CNV results were consistent with the results obtained from pathological identification, and tumour samples displayed high CNV scores (Figure 3B,C). However, the tumour samples showed distinct patterns, which suggested the presence of intratumoral genomic heterogeneity.

After extracting gene expression data from each sample, we analyzed differentially expressed genes (DEGs) specific to normal pituitary and tumour tissues and then, filtered the DEGs by calculating the Δ percentage difference.^33^ The top 10 upregulated or downregulated DEGs with Δ percentage difference >20% were labelled (Figure 3D). The expression of EIF1AY and KDM5D was elevated in tumour samples but almost absent in normal pituitary tissue. LHB and PGR exhibited high expression levels in normal samples and paratumoural regions of tumour samples; however, their expression levels were relatively low in the tumour region of tumour samples (Figure 3F and Figure S9). The above results were validated by immunohistochemistry. Additionally, Gene Ontology (GO) enrichment analyses and Kyoto Encyclopedia of Genes and Genomes (KEGG) analyses were performed on DEGs that were upregulated in tumour samples compared to their expression in normal pituitary. These upregulated DEGs were mainly enriched in processes such as inflammatory response, cell adhesion and positive regulation of cell proliferation (Figure 3E).

As the proportion of epithelial cells was the highest in all samples, we further assessed the differences in spatial distribution and DEGs of epithelial cells between tumour and normal tissues (Figure 3G and Figure S4A). In the epithelial cell area of ​​tumour tissues, six clusters with significantly different levels of CNVs were obtained. Among them, cluster 5 had the highest level of CNVs, and PIGR, THBS2 and LCN2 were its marker genes (Figure 3H‐J). In the epithelial cell region of normal tissues, five clusters were obtained (Figure 3K,L). Additionally, fibroblasts help maintain tissue homeostasis and are an important component of the TME.^34^ To investigate the features of fibroblasts, we determined their distribution (Figure S4B). Then unbiased clusters and marker genes in fibroblasts of tumour or normal tissues were obtained (Figure S4C). Our results indicated the high heterogeneity in epithelial cells and fibroblasts of tumour tissues.

### 2.4 Intratumoral heterogeneity of somatotroph PitNETs revealed by spatial transcriptomics from the perspective of CNVs

**FIGURE 4:**
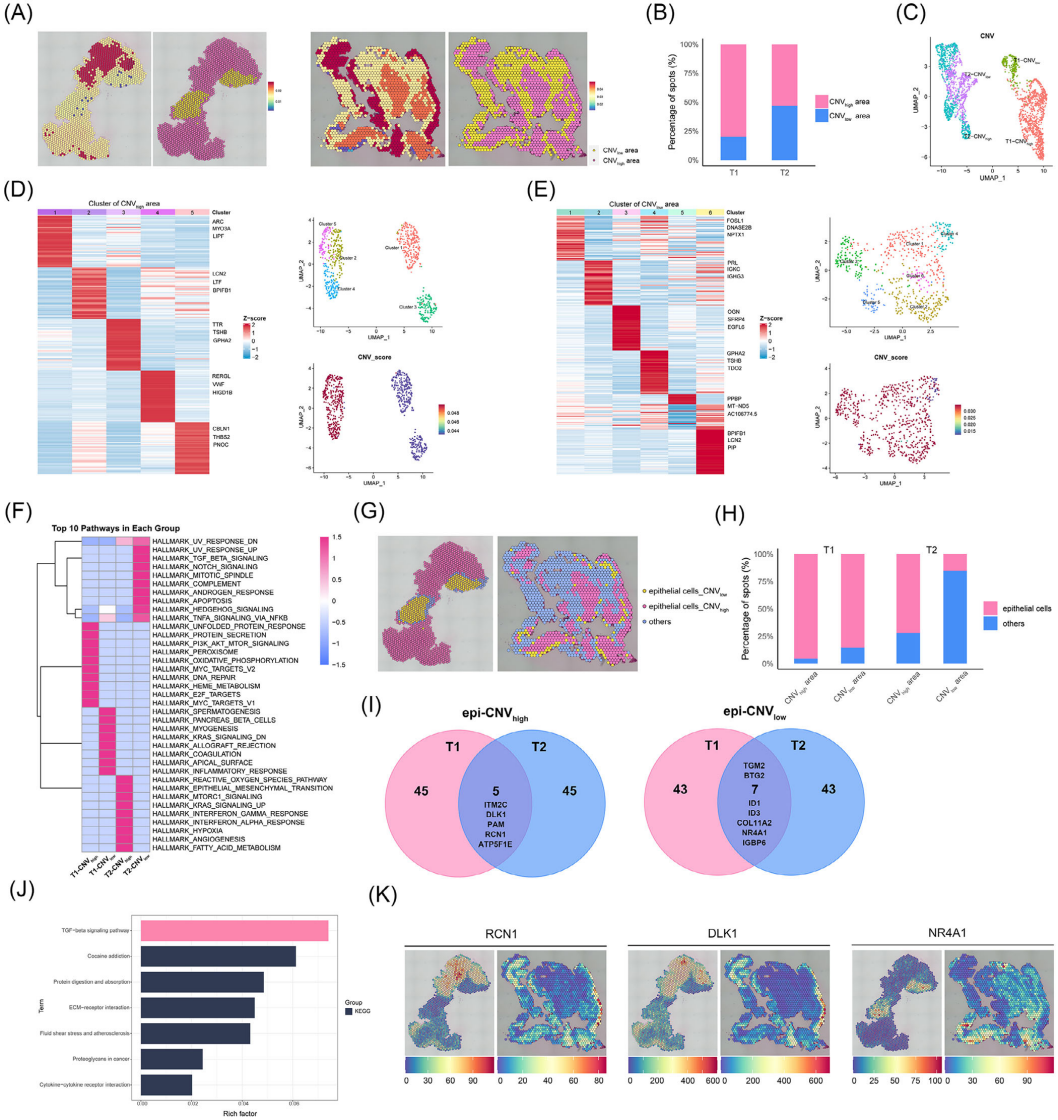
Spatial transcriptome signatures and heterogeneity in somatotroph tumour tissues. (A) The spatial spots are coloured based on the copy number variation (CNV) levels (left) and divided into the CNV_high_ area or the CNV_low_ area (right). (B) The bar plot shows the percentage of CNV_high_ area and CNV_low_ area in tumour samples. (C) The UMAP plot depicts spots in tumour tissues, coloured based on samples and CNV levels. (D) A heatmap of the cluster‐specific genes of the CNV_high_ area in the T2 sample. The UMAP plot shows spots of CNV_high_ area in the T2 sample, colored based on unbiased clustering and CNV levels. (E) A heatmap of the cluster‐specific genes of the CNV_low_ area in the T2 sample. The UMAP plot depicts unbiased clustering of the CNV_low_ area in the T2 sample. (F) A heatmap of the top 10 GSVA enrichment pathways for tumour and paratumour regions in each tumour sample. (G) The spatial distribution of epi‐CNV_high_ and epi‐CNV_low_ areas in the T1 and T2 samples. (H) The bar plot indicates the proportion of epithelial cells divided by the CNV_high_ and CNV_low_ areas. (I) The DEGs of epi‐CNV_high_ and epi‐CNV_low_ were shared by the T1 and T2 samples. (J) Significant KEGG terms of epithelial cells in the epi‐CNV_high_ group compared to the epi‐CNV_low_ group in the T1 and T2 samples. (K) The spatial distribution of the RCN1, DLK1 and NR4A1 genes. epi‐CNV_high_, epithelial cells with high CNV levels; epi‐CNV_low_, epithelial cells with low CNV levels.

To plot the internal map of tumour tissues, we used two approaches to identify the tumour and paratumour regions. One approach was pathological identification, whereas the other approach involved implementing the inferCNV methods to infer CNVs from spatial transcriptome data and label CNV_high_ and CNV_low_ regions based on CNV scores (Figure S5A). The results matched the approximate areas of the tumour and paratumour identified by pathologists (Figure 4A). The proportion of CNV_high_ and CNV_low_ regions and the level of CNVs were different, indicating high intertumor heterogeneity (Figures 4B,C). Thus, the T1 and T2 samples were analyzed separately (Figure 4D,E and Figure S5B). Unsupervised clustering was performed, and the level of CNVs was determined in the CNV_high_ and CNV_low_ regions of the T2 sample (Figure 4D,E). The CNVs showed higher heterogeneity within the CNV_high_ region. Next, GSVA was performed according to the CNV_high_ and CNV_low_ regions, and the results showed significant differences between different samples and different regions (Figure 4F).

Epithelial cells constituted a significant proportion of tumours, and thus, we divided epithelial cells into CNV_high_ and CNV_low_ (epi‐CNV_high_ and epi‐CNV_low_) regions (Figure 4G). As shown in the bar graph (Figure 4H), the proportion of epithelial cells in the CNV_high_ region was higher. The DEGs of epi‐CNV_high_ and epi‐CNV_low_ were further analyzed in T1 and T2 samples. The top 50 DEGs of T1 and T2 samples were intersected, and the results showed that the ITM2C, DLK1, PAM, RCN1 and ATP5F1E genes were highly expressed in the epi‐CNV_high_ region, while the TGM2, BTG2, ID1, ID3, COL11A2, NR4A1 and IGBP6 genes were enriched in the epi‐CNV_low_ region (Figure 4I). The spatial distribution of RCN1, DLK and NR4A1 is shown in Figure 4K. Subsequently, migration assays were performed to assess the effect of DLK1 and RCN1 on cell migration (Figures S6 and S7). Transwell assays demonstrated that DLK1 and RCN1 promoted the invasion of pituitary tumour cells. Immunohistochemistry assays provided further confirmation of the high expression of DLK1 and RCN1 in tumour tissues, suggesting that they were associated with tumour progression (Figure S8A–C). The results of the KEGG enrichment analysis showed that the epithelial cells of T1 and T2 were closely related to the TGF‐β signalling pathway (Figure 4J). These findings collectively supported the high intertumoral and intratumoral heterogeneity of somatotroph PitNETs from the perspective of CNVs.

### 2.5 Single‐cell transcriptomic atlas of somatotroph PitNETs and normal pituitary

**FIGURE 5:**
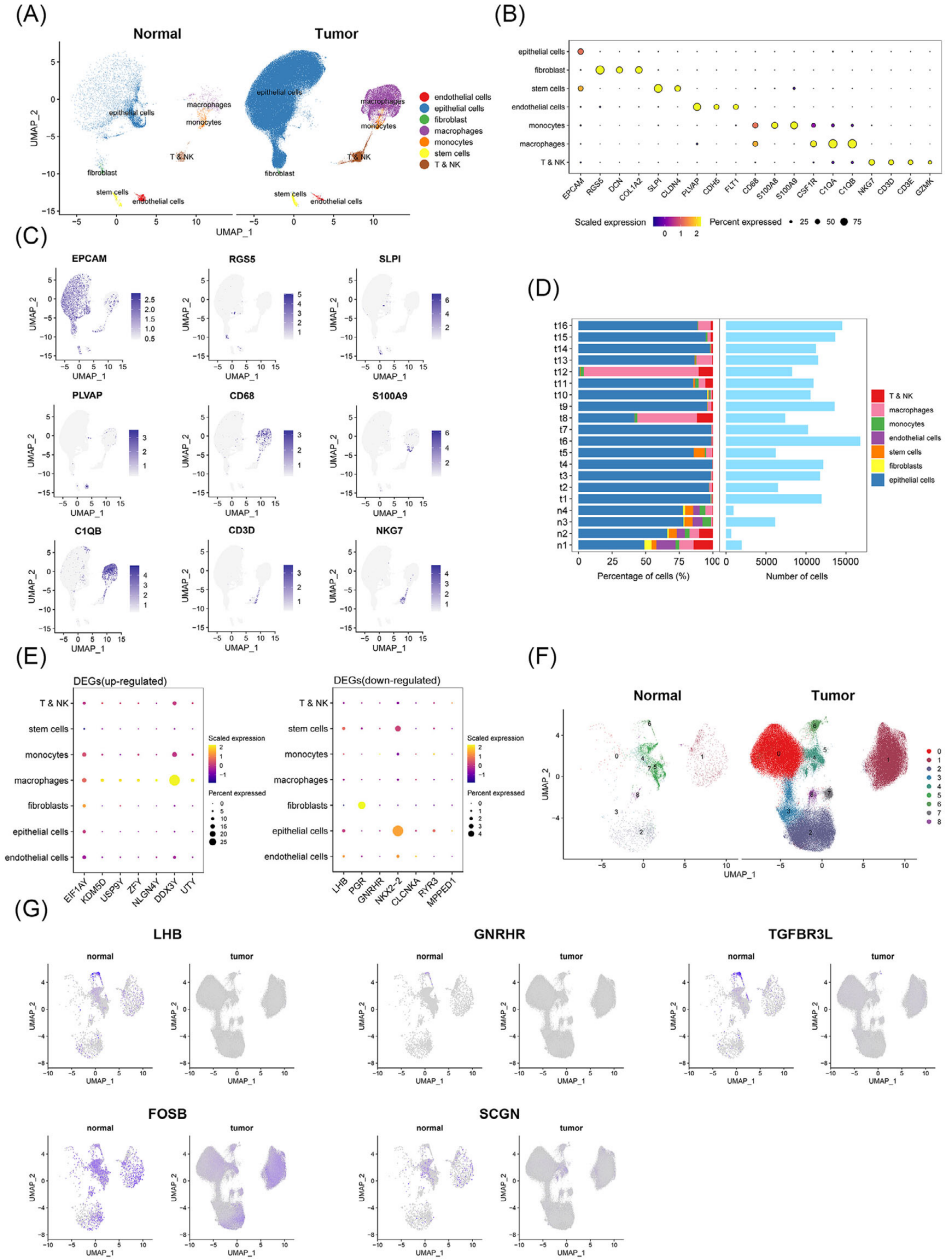
Single‐cell transcriptome characterization of somatotroph tumours and the normal pituitary. (A) UMAP plots of 9679 cells obtained from the normal pituitary and 177,213 cells obtained from somatotroph tumour tissues. Seven clusters are shown in each plot. Each cluster is represented using a different colour. (B) The dot plots show the average expression of marker genes in the indicated cell cluster. The dot size represents the percentage of cells expressing the gene in each cluster and the intensity of expression of the marker gene is shown. (C) The UMAP plots of the normal pituitary and tumour tissues illustrate the level of expression of selected markers across 186,892 unsorted cells. (D) The bar plots show the proportion of seven major cell types and the total cell count in different samples. (E) The dot plot displays the expression of DEGs in different cell types in the scRNA‐seq data. The DEGs were screened based on the comparison of the normal and tumour groups in the spatial transcriptome data. (F) UMAP plots of epithelial cells in the normal and tumour groups. (G) UMAP plots of selected DEGs. These DEGs were screened by comparing epithelial cells of the normal and tumour groups obtained from the spatial transcriptome data.

To provide more information on the spatial distribution and cellular characteristics of somatotroph tumours and normal pituitary, we collected scRNA‐seq data from four normal pituitary samples (n1–n4) and 16 somatotroph tumours (t1–t16) for auxiliary analysis. The main cell types were the same as those in the spatial transcriptome data; however, immune cells, especially macrophages, were significantly more abundant in the scRNA‐seq data (Figure 5A). These cells were classified into seven major cell types, including epithelial cells (EPCAM), fibroblasts (RGS5, DCN and COL1A2), stem cells (EPCAM, SLPI and CLDN4), endothelial cells (PLVAP, CDH5 and FLT1), monocytes (CD68, S100A8 and S100A9), macrophages (CD68, CSF1R, C1QA and C1QB), and T & NK cells (NKG7, CD3D, CD3E and GZMK) (Figure 5B). UMAP plots were constructed to visualize the distribution of major marker genes (Figure 5C). In the bar plot, we found that except for the t12 sample, which was dominated by macrophages, the other samples were dominated by epithelial cells. The types of cells were more abundant and similar in normal pituitary, but the proportion of different cell types in tumours varied greatly (Figure 5D). The spatial transcriptome data identified differential expression of DDX3Y, PGR and NKX2‐2 between tumour samples and normal samples (Figure 3D). The scRNA‐seq data showed that DDX3Y was mainly expressed in macrophages, PGR was mainly expressed in fibroblasts, and NKX2‐2 was predominantly expressed in epithelial cells (Figure 5E). Epithelial cells were extracted separately, and the epithelial cells in the normal group were dominated by cluster 5 (Figure 5F). The UMAP plots showed that the LHB, GNRHR, TGFBR3L, FOSB and SCGN genes were highly expressed mainly in the epithelial cells of the normal group, while their expression was low in the epithelial cells of the tumour group (Figure 5G). These results are similar to the spatial transcriptome data (Figure S4A). Additionally, the largest number of myeloid cell populations (macrophage and monocyte) caught our attention (Figure 5A). These cell populations were analyzed for more subdivision, annotation and function enrichment analysis (Figure S10). Interestingly, the proportion of the Mac_MEG3 cluster in tumour samples was found significantly increased, while Mac_FCN1 and Mac_CCL3 clusters were significantly dominant in normal samples.

### 2.6 Transcriptomic heterogeneity in epithelial cells of somatotroph PitNETs

**FIGURE 6:**
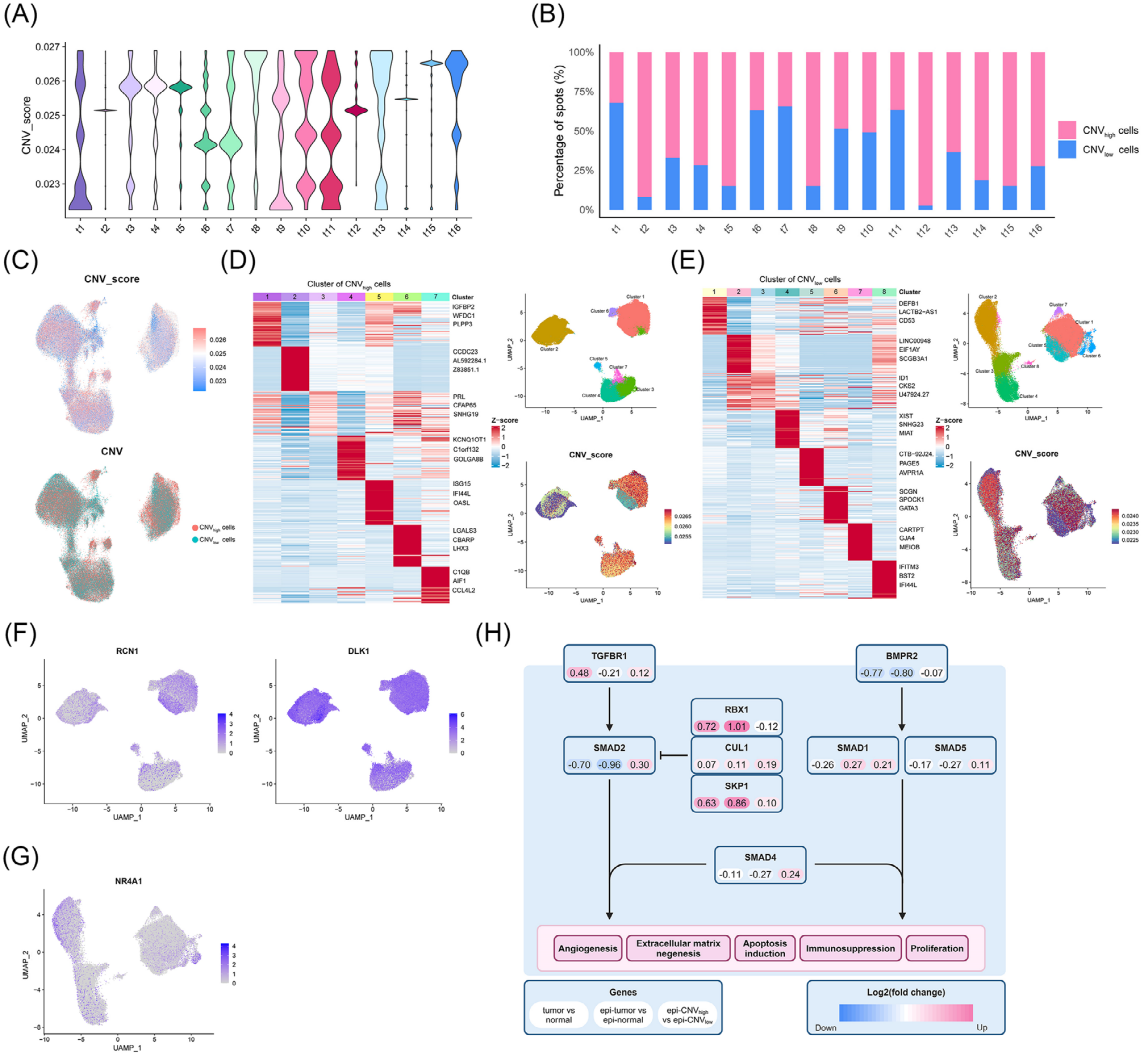
Single‐cell transcriptome heterogeneity in epithelial cells of somatotroph pituitary neuroendocrine tumours (PitNETs). (A) A violin plot of the copy number variation (CNV) scores of each tumour sample. (B) The bar graph shows the proportion of epi‐CNV_high_ cells and epi‐CNV_low_ cells in tumour samples. (C) The UMAP plots are coloured based on the CNV score and the epi‐CNV_high_ or epi‐CNV_low_ cells. (D) A heatmap of the cluster‐specific genes of epi‐CNV_high_ cells. The UMAP plots show unbiased clustering and CNV scores. (E) The epi‐CNV_low_ cells are presented using the same representation as epi‐CNV_high_ cells. (F) The UMAP plots show the distribution of RCN1 and DLK1 genes in epi‐CNV_high_ cells. (G) The UMAP plot shows the distribution of the NR4A1 gene in epi‐CNV_low_ cells. (H) The diagram summarizes the genes associated with the TGF‐β signalling pathway. Alterations were expressed by upregulation and downregulation of mRNA expression as follows: tumour versus normal (left), epi‐tumour versus epi‐normal (middle) and epi‐CNV_high_ versus epi‐CNV_low_ (right). The alteration score for each gene was presented as log2(fold change). Colour: red (upregulation) and blue (downregulation).

To investigate the neoplastic development and progression of somatotroph PitNETs facilitated by the different epithelial cells enriched in tumour tissues, we performed a CNV analysis of all epithelial cells in the tumour group obtained from the scRNA data (Figure 6A). We identified epi‐CNV_high_ and epi‐CNV_low_ cells based on the level of CNVs present (Figure 6B,C). These results suggested that the proportion of epi‐CNV_high_ was different in different tumour samples, which indicated differences in the degree of epi‐CNV_high_ infiltration within tumour tissues. Unsupervised clustering was performed in the epi‐CNV_high_ and epi‐CNV_low_ groups, and the marker genes and CNV scores across the clusters are shown in Figure 6D,E. We examined the scRNA‐seq distribution of the differentially expressed genes RCN1 and DLK1, which were highly expressed in epi‐CNV_high_ and NR4A1 in epi‐CNV_low_ found in the spatial transcriptome (Figures 4K and 6F,G). Since the TGF‐β signalling pathway was enriched in epithelial cells, we further analyzed the changes in the genes within this signalling pathway in the scRNA‐seq data (Figures 4J and 6H). The results demonstrated that the expression of genes within the TGF‐β signalling pathway exhibited heterogeneity in somatotroph tumours. The heterogeneity of the TGF‐β signalling pathway in somatotroph pituitary tumours was verified by immunohistochemistry assays (Figure S8D). The results demonstrated that SKP1 was highly expressed in tumour tissues, whereas Smad2 and BMPR2 were inhibited, which was consistent with the findings of scRNA‐seq data. In conclusion, the results of the CNV analysis based on scRNA‐seq demonstrated that the tumour epithelial cells were heterogeneous.

CellChat2 was applied to all epithelial cells and myeloid cell subsets to explore cell‐to‐cell communication in the pituitary tumour microenvironment. A brief analysis of the differences in communication between epithelial cells and myeloid cells between pituitary tumours and normal samples was performed. The number and intensity of inferred interactions in tumour samples were found to be higher than in normal samples (Figure S11A), and there were numerous pathways with significant differences between them (Figure S11B). Notably, the GH signalling pathway was found to be highly active in tumour groups. The interaction network indicates that Mac_MEG3 is the primary signaler of this pathway in macrophages (Figure S11D), suggesting that the secretion of GH is closely related to this cell population. These above results suggest a unique pattern of immune response in TME of somatotroph pituitary tumours.

## 3 DISCUSSION

Tumor heterogeneity is the primary determinant of tumour progression and treatment efficacy.^35^, ^36^ PitNETs are characterized by high tumour heterogeneity. However, information on the spatial heterogeneity of PitNETs is extremely limited. We used ST and scRNA‐seq technologies to characterize spatial transcriptome profiles of somatotroph tumours and the normal pituitary to comprehensively understand the mechanism of tumour development and invasion in this study.

Initially, we constructed a spatial landscape of cellular distribution in the normal pituitary and somatotroph tumours. Epithelial cells are the most predominant type of cells in tumours. Therefore, we investigated the heterogeneity of gene expression signatures in epithelial cells. The TGF‐β signalling pathway was enriched, and the key genes were found to be heterogeneous in spatial distribution. As the intratumoral heterogeneity of epithelial cells may contribute to treatment failure, elucidating the spatial distribution of epithelial cells may facilitate individualized precision therapy. Moreover, non‐tumour cells, such as fibroblasts, immune cells and endothelial cells, are important components of TME, which also determine the heterogeneity of TME.^36^, ^37^, ^38^ Fibroblasts are important for normal tissue homeostasis, but tumour‐associated fibroblasts (TAFs) are potential targets of anti‐tumour therapy.^15^, ^17^ TAF‐derived cytokines in somatotroph PitNETs increase the aggressiveness of tumours, which can be inhibited by the anti‐tumour effects of pasireotide.^39^ TAF‐derived exosomal circDennd1b promotes the progression of PitNETs by regulating the miR‐145‐5p/ONECUT2 axis.^40^ In a study, scRNA‐seq validated the key role of IFN‐γ‐induced remodelling of TAFs in the progression of PitNETs.^15^ This was the first study in which fibroblasts in tumour tissue and normal tissue were spatially visualized. However, analyzing fibroblasts from the perspective of spatial transcriptome data requires further assessment. We also found that the immune function in the paratumour region was more active, and further analysis needs to be performed for a more comprehensive understanding of the data.

Along with pathologist annotations, the CNV analysis could more accurately describe the malignant degree of tumour cells. Different studies have found different extents of genomic heterogeneity in PitNETs.^14^, ^41^, ^42^ Our findings provided new insights into the genomic heterogeneity of pituitary tumours. We also found differences in the expression of genes and functional heterogeneity in the CNV_high_ and CNV_low_ regions of tumour samples. Additionally, our results showed that the genes DLK1 and RCN1 may be associated with the progression of somatotroph PitNETs. The expression of DLK1 is abnormal in malignant tumours,^43^ including glioblastoma,^44^ lung cancer,^45^ and ovarian cancer^46^ and it can promote invasion of lung cancer cells by upregulating MMP9 expression levels.^47^ However, further studies are needed to determine the roles of DLK1 and RCN1 in PitNETs. In the future, we aim to investigate the mechanism underlying the functions of these genes in PitNETs. In this study, we reported the presence of intertumoral and intratumoral spatial heterogeneity; thus, emphasizing the importance of focusing on individual profiles.

Finally, we initially collected many FFPE samples of somatotroph PitNETs in this study. In order to study the tumour and paratumour regions in the tumour samples, as well as the normal pituitary sample characteristics, we finally selected four precious finite FFPE samples for subsequent ST. The Visium CytAssist ST technique was a mature and appropriate technique for processing those FFPE samples. However, this study had some limitations. The FFPE samples were sequenced for ST, which was prone to loss of immune cells. Therefore, we did not investigate immune cells in this study. As PitNETs exhibited high heterogeneity, we relied on spatial transcriptome data rather than single‐cell data for marker annotation. Additionally, the sample size of this study is small and hard to increase due to the difficulty in collecting suitable samples. Although single‐cell data are available for auxiliary analysis, a larger cohort is needed for subsequent confirmation.

To summarize, in this study, we characterized and revealed the cellular characterization of somatotroph tumours and normal pituitary. This study represents the first application of ST in somatotroph PitNETs. Our results showed intertumoral and intratumoral heterogeneity of PitNETs and the unique gene expression patterns and functions in different regions of epithelial cells. These differences can be used to identify regulatory mechanisms and therapeutic targets. The spatial information obtained from this study might help in developing effective targeted therapeutic strategies for treating PitNETs.

## 4 MATERIALS AND METHODS

### 4.1 Patients and clinical samples

All patients provided written informed consent before this study was conducted. All procedures were approved by the Institutional Review Board of Beijing Tiantan Hospital, Capital Medical University (No. KY 2018‐053‐02).

For ST sequencing, formalin‐fixed paraffin‐embedded (FFPE) tissues of normal pituitary and somatotroph PitNET cases were collected from four patients who underwent pituitary surgery at Beijing Tiantan Hospital from 2022 to 2023. Four normal pituitary samples and 16 somatotroph PitNET cases were used for scRNA‐seq analysis; the analyses were performed based on new scRNA‐seq data and published data (Table S1).^13^ The normal pituitary tissues were obtained by endonasal endoscopy, which required partial pituitary resection and transposition. All diagnoses of somatotroph PitNETs were confirmed by a multidisciplinary team consisting of neurosurgeons, neuropathologists, and neuroradiologists. Somatotroph PitNETs in this study were diagnosed based on the clinical manifestations consistent with acromegaly^48^ and it was ensured that they met the diagnostic criteria for somatotroph tumours established by the World Health Organization (2022).^49^ Note that all data analysis and experiments were performed with unknown sample ID information.

### 4.2 Sample preparation for ST

ST on FFPE slides was performed using 10X Genomics Visium CytAssist Spatial Gene Expression for FFPE tissues (CG000495). First, 2–3 consecutive tissue sections (5‐µm thick) were obtained for RNA extraction using a Qiagen RNeasy FFPE kit. Purified RNA was used to evaluate the quality of RNA in tissues. DV200 was calculated using Agilent RNA 6000 Pico kit and blocks with DV200 > 30% were chosen for further sectioning. The tissue sections were deparaffinized and then stained with H&E. The sections were subjected to coverslipping, imaging and decoverslipping and subsequently underwent decrosslinking. A human whole transcriptome probe panel was used to deparaffinized, stain and decrosslinked tissue. Following probe hybridization and ligation, tissue sections were processed utilizing a Visium CytAssist instrument. The analytes were transferred to the Visium CytAssist spatial gene expression slides, which have a capture area of 6.5 × 6.5 mm. After RNA digestion and tissue removal, single‐stranded probe ligation products were released from FFPE tissues and then the products were captured on Visium slides. UMIs, spatial barcodes and partial Illumina read 1 were combined to extend probes. Extended probes were used to generate the libraries, which were then quantified and assessed for quality using the Qubit and Agilent TapeStation. Finally, the libraries were sequenced on the Illumina NovaSeq 6000 System.

### 4.3 Analysis of ST data

After sequencing, the raw files were processed using Space Ranger 2.0.1. Demultiplex the raw base sequence calls to provide the FASTQ files using bcl2fastq. FASTQ files were then subjected to sequence alignment, tissue detection, fiducial detection and spatial barcode/UMI counting using spaceranger count. Reads were aligned to the human genome reference sequence (GRCh38) and human whole transcriptome probe set (Visium_Human_Transcriptome_Probe_Set_v2.0_GRCh38‐2020‐A.csv). The reads that were aligned to the probe set were then assigned to the respective genes and mapped to the specific spots based on the spatial barcodes. The expression of genes was quantified based on the number of UMI detected within each spot. Data were analyzed using the R package Seurat 4.3.0 after preprocessing with spaceranger.^50^, ^51^, ^52^ Sctransform was used to normalize the spatial transcriptome dataset. Markers for each identified cluster were found using the FindAllMarkers function in Seurat. Finally, based on the expression of canonical markers of specific cell types, the clusters were classified and annotated.

### 4.4 Sample preparation for scRNA‐seq

Initially, fresh somatroph PitNET tissues were washed with phosphate‐buffered saline (PBS) and minced into small pieces. Then, tissues were incubated with a digestion solution containing PBS and 1.5 mg/mL collagenase II and IV (Gibco) at 37°C and 800 rpm for 30 min. The mixture was filtered through a 45‐µm nylon mesh and digested again using accutase to prepare a single‐cell suspension.

### 4.5 Processing of scRNA‐seq data, cluster annotation and data integration

The analyses were performed using the Seurat package (v 4.3.0) and R (v 4.2.2). We regressed out the following confounding factors: number of UMIs, patient ID, percentage of mitochondrial RNA and cell cycle (S and G2 M phase scores calculated using the CellCycleScoring function in Seurat) after clustering the cell types. Robust principal component analysis (RPCA) was performed for sample integration, after which UMAP was used for dimension reduction. After clustering, we identified and removed clusters containing low‐quality and doublet cells and then reclustered the remaining cells. We used the FindMarkers function to determine highly expressed genes for each cluster. The resulting clusters were annotated to cell types based on marker gene expression levels.

### 4.6 Sample correlation analysis

The Spearman correlation was used to calculate the correlation between the samples, and the samples were hierarchically clustered using the differential genes Nomex Pumatrix (Linial model) and the obtained corresponding Pusado Bourk Nomex Pumatrix.^53^

### 4.7 Immune score analysis

For ST data, the immune scores of spatial spots were calculated by the AddModuleScore function with the parameter (crtl = 100) in Seurat using an immune gene list. The list of genes is: “PTPRC”, “CD3D”, “CD3E”, “CD4”, “CD8A”, “GZMK”, “NKG7”, “KLRD1”, “CD79A”, “IGHG1”, “MS4A1”, “CD68”, “LYZ”, “S100A8”, “S100A9”, “CSF1R”, “C1QA”, “C1QB”, “CD1C”, “IL3RA”, “LILRA4” and “IRF7”.

### 4.8 Identification and functional enrichment of DEGs

The FindAllMarkers function in Seurat (v 4.3.0) was used to identify markers for each cluster.^51^ Subsequently, the clusters were characterized and annotated based on the expression of canonical markers of specific cell types. The FindMarkers function was used to identify DEGs between two groups of cells using the default parameters (logfc.threshold = 0.25, test.use = “wilcox”, min.pct = 0.1), and the results were further filtered based on an adjusted *p*‐value of  .01. The differentially expressed gene sets were annotated using the clusterProfiler package (v.4.6.2) with the GO and KEGG databases.^54^ Enrichment pathways were obtained with *p*.adjust < .05, and *p*‐value adjustment (FDR) was performed by Benjamini–Hochberg method. Gene set variation analysis (GSVA) was performed using the GSVA package v.1.50.0.^55^

### 4.9 CNV estimation in PitNETs

The Infercnvpy package (v 0.4.1) was used to infer CNVs in the spatial transcriptome data and single‐cell transcriptome data using default parameters. The CNV signal for individual cells was estimated with a 50‐gene sliding window. For tumour samples, CNV was inferred from scRNA‐seq using inferCNV with normal epithelial cells as a control. Based on the CNV score distribution, the cells were divided into the CNV_low_ group and the CNV_high_ group. For scRNA‐seq data and ST data, the thresholds were set to the median.

### 4.10 Data and code availability

The raw sequence data reported in this paper have been deposited in the Genome Sequence Archive (Genomics, Proteomics & Bioinformatics 2021) in National Genomics Data Center (Nucleic Acids Res 2022), China National Center for Bioinformation/Beijing Institute of Genomics, Chinese Academy of Sciences (GSA‐Human: HRA007285) that are available at https://bigd.big.ac.cn/gsa‐human/browse/HRA007285. We did not generate original codes. All software and algorithms used in this study are publicly available.

## AUTHOR CONTRIBUTIONS

**Jialin Wang**: Prepared manuscript writing and figures and contributed to the study design and manuscript revision. **Xuejing Li**: Conducted histopathological annotation of samples. **Jing Guo**: Contributed to sample collection and clinical data management. **Zan Yuan and Xinyu Tong**: Contributed to figure preparation and bioinformatics analyses. **Zehao Xiao**: Contributed to sample collection. **Meng Liu and Changxiaofeng Liu**: Contributed to bioinformatics analyses. **Hongyun Wang and Lei Gong**: Contributed to the management of specimens and clinical data. **Chuzhong Li and Yazhuo Zhang**: Contributed to the supervision. **Weiyan Xie and Chunhui Liu**: contributed to the sample preparation; study design; critical revision and funding acquisition. All authors approved the final manuscript.

## CONFLICT OF INTEREST STATEMENT

The authors declare no conflict of interest.

## ETHICS STATEMENT

All patients provided written informed consent before this study was conducted. All procedures were approved by the Institutional Review Board of Beijing Tiantan Hospital, Capital Medical University (No. KY 2018‐053‐02).

## Supporting information



Supporting Information

Supporting Information

Supporting Information

Supporting Information

Supporting Information

Supporting Information

Supporting Information

Supporting Information

Supporting Information

Supporting Information

Supporting Information

Supporting Information
