# Supplementary figures and images for: Comprehensive mapping of somatotroph pituitary neuroendocrine tumour heterogeneity using spatial and single‐cell transcriptomics
Source: Clin Transl Med. 2024 Nov 15;14(11):e70090. doi: 10.1002/ctm2.70090 (PMC11567828; doi:10.1002/ctm2.70090)

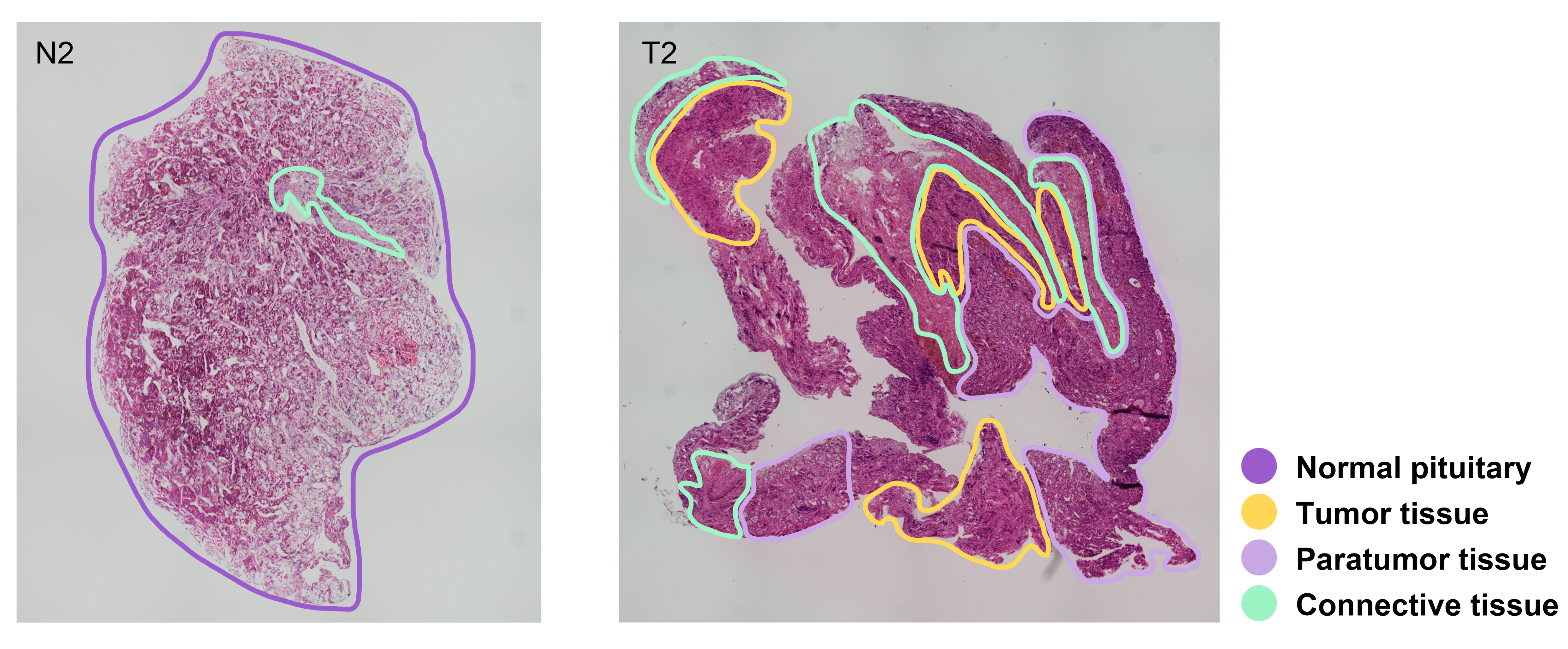

Supplement: Supplementary file 1 — Supporting Information [file CTM2-14-e70090-s001.tif]

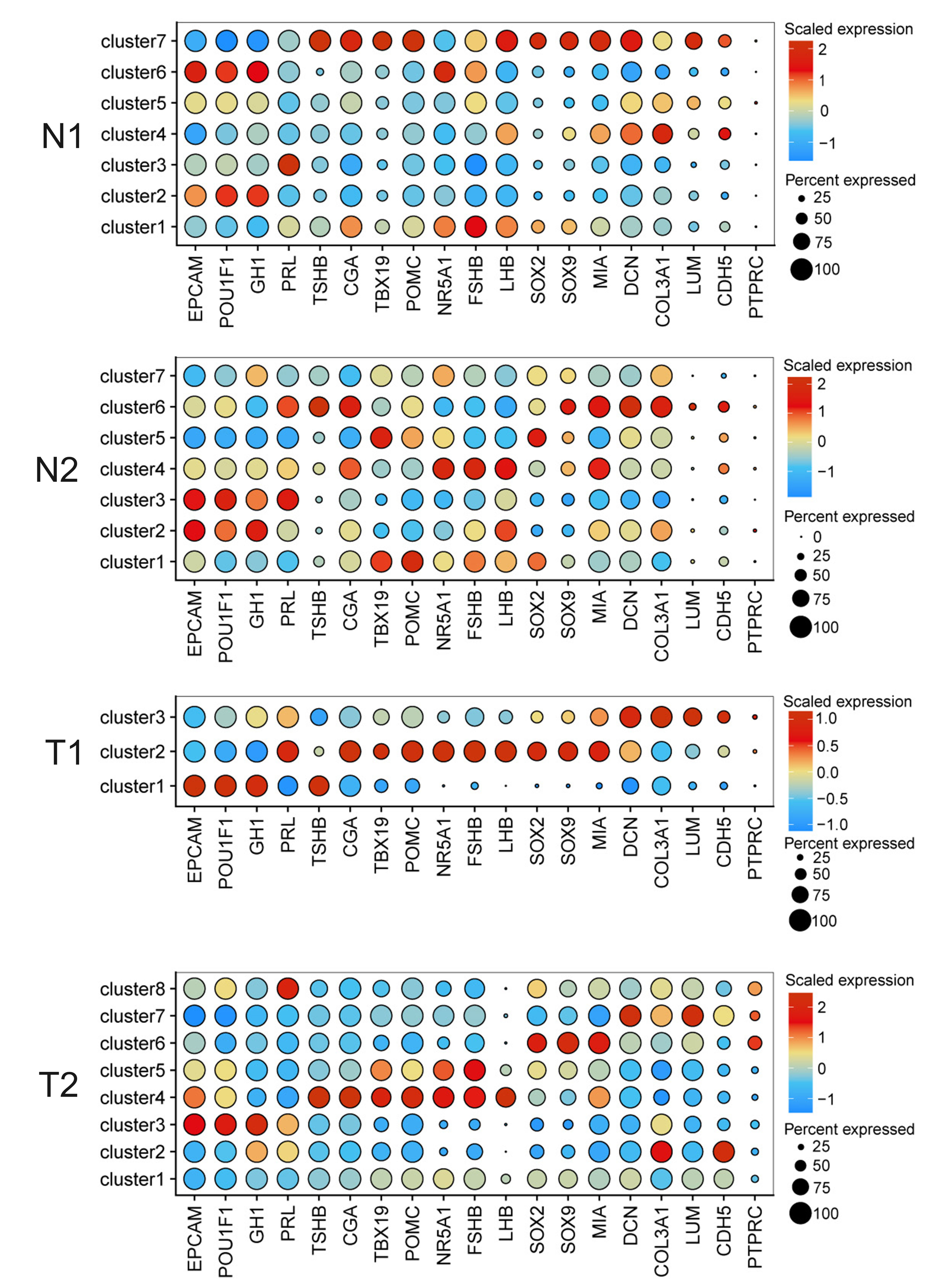

Supplement: Supplementary file 2 — Supporting Information [file CTM2-14-e70090-s010.tif]

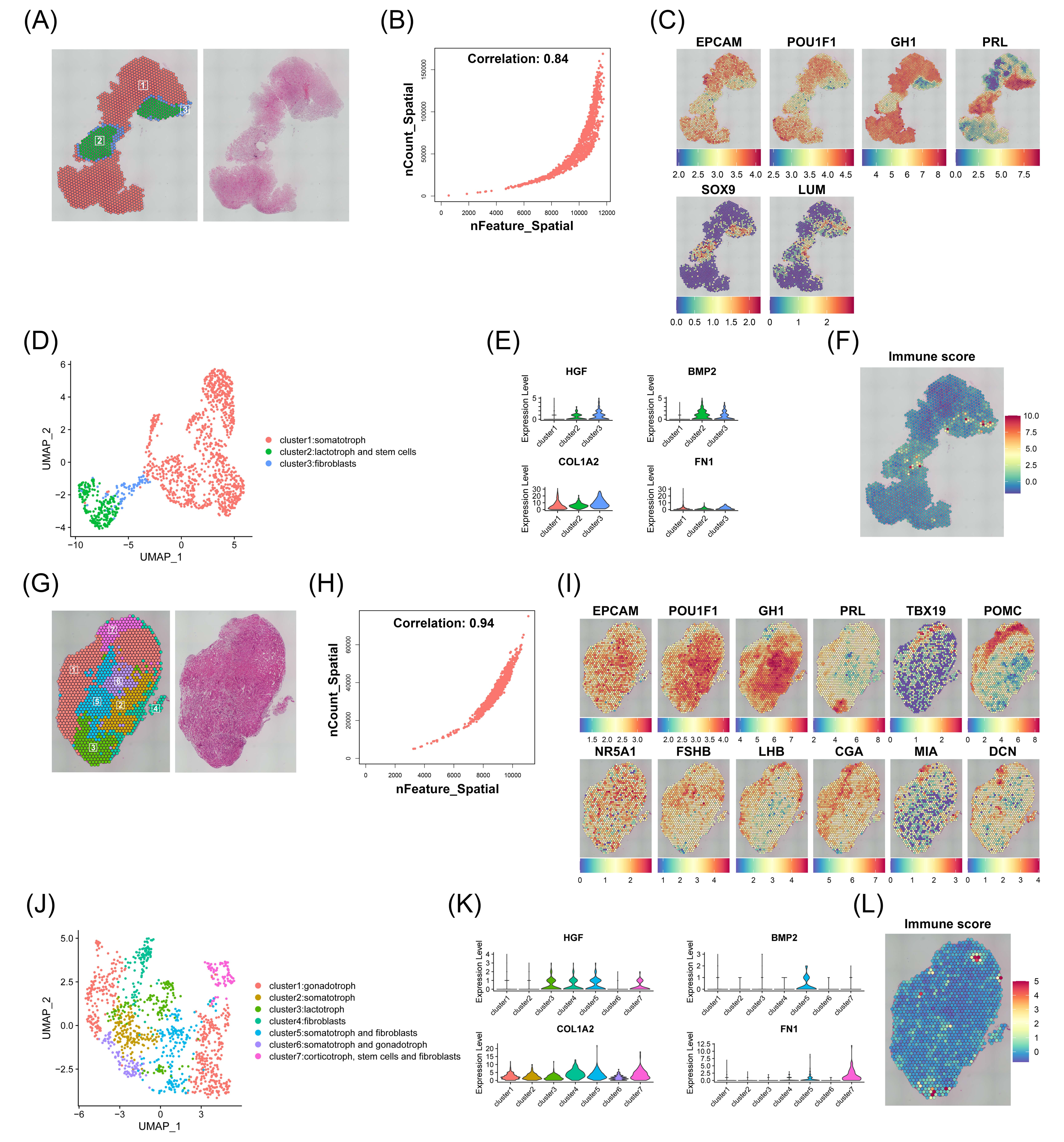

Supplement: Supplementary file 3 — Supporting Information [file CTM2-14-e70090-s005.tif]

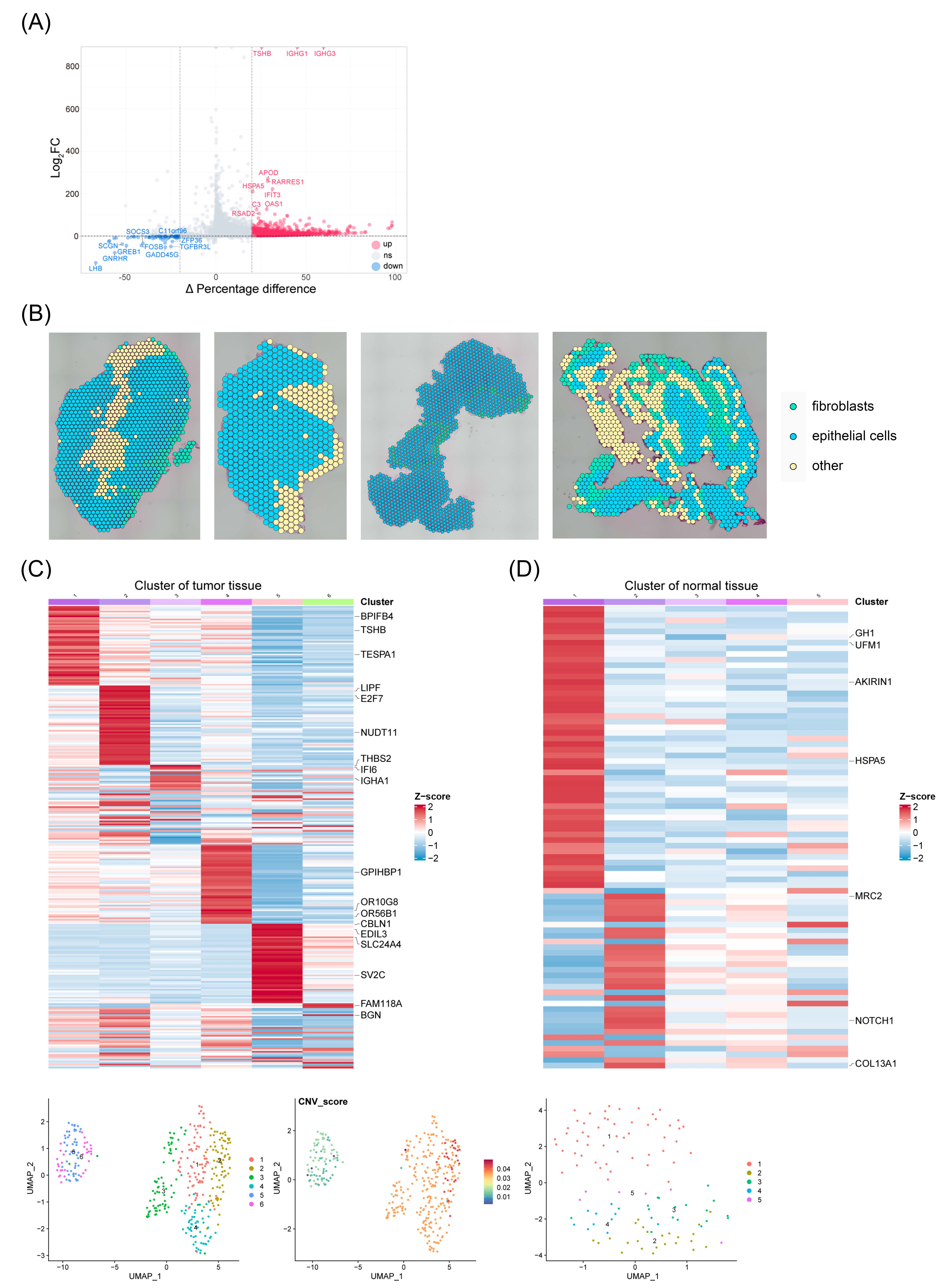

Supplement: Supplementary file 4 — Supporting Information [file CTM2-14-e70090-s006.tif]

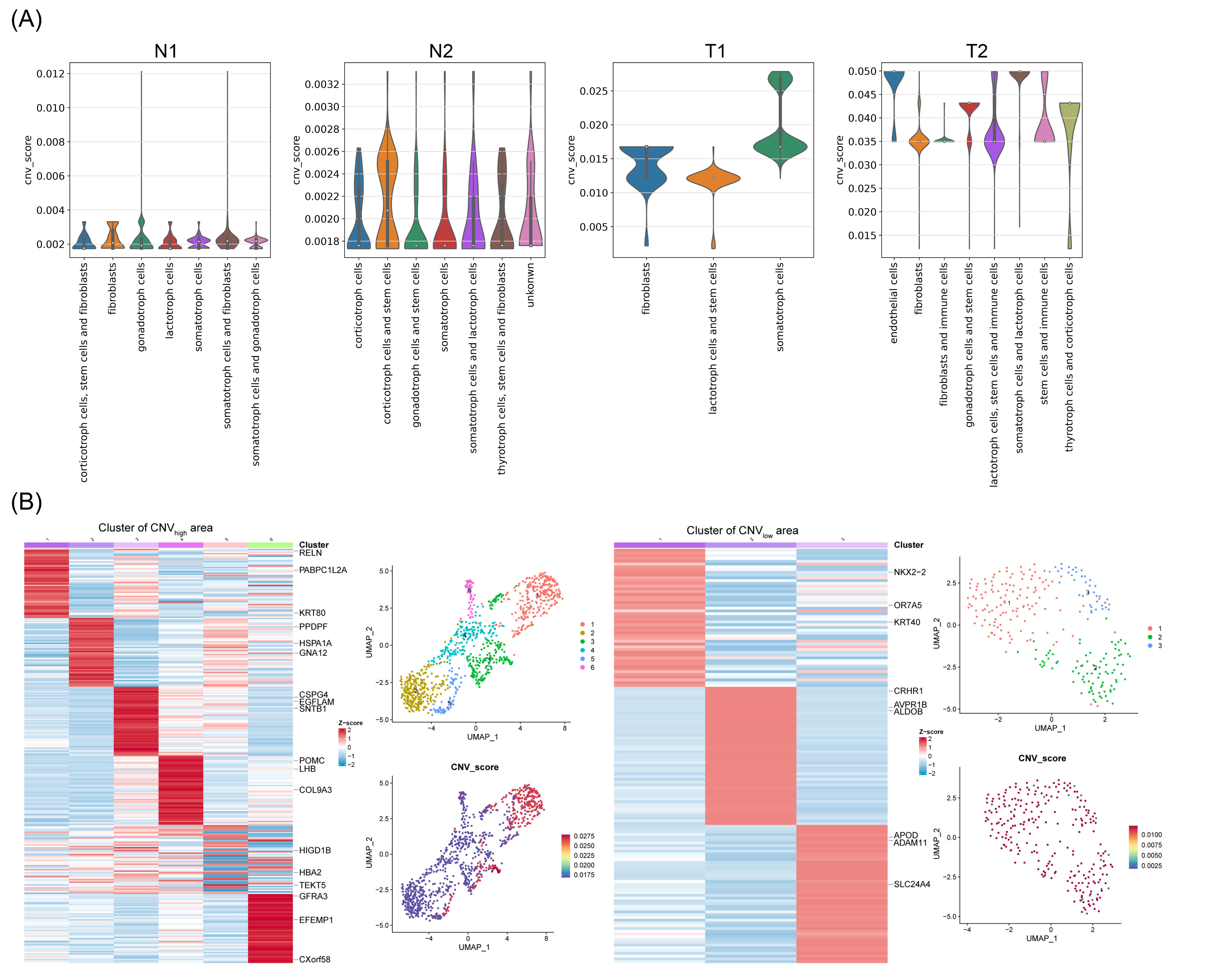

Supplement: Supplementary file 5 — Supporting Information [file CTM2-14-e70090-s008.tif]

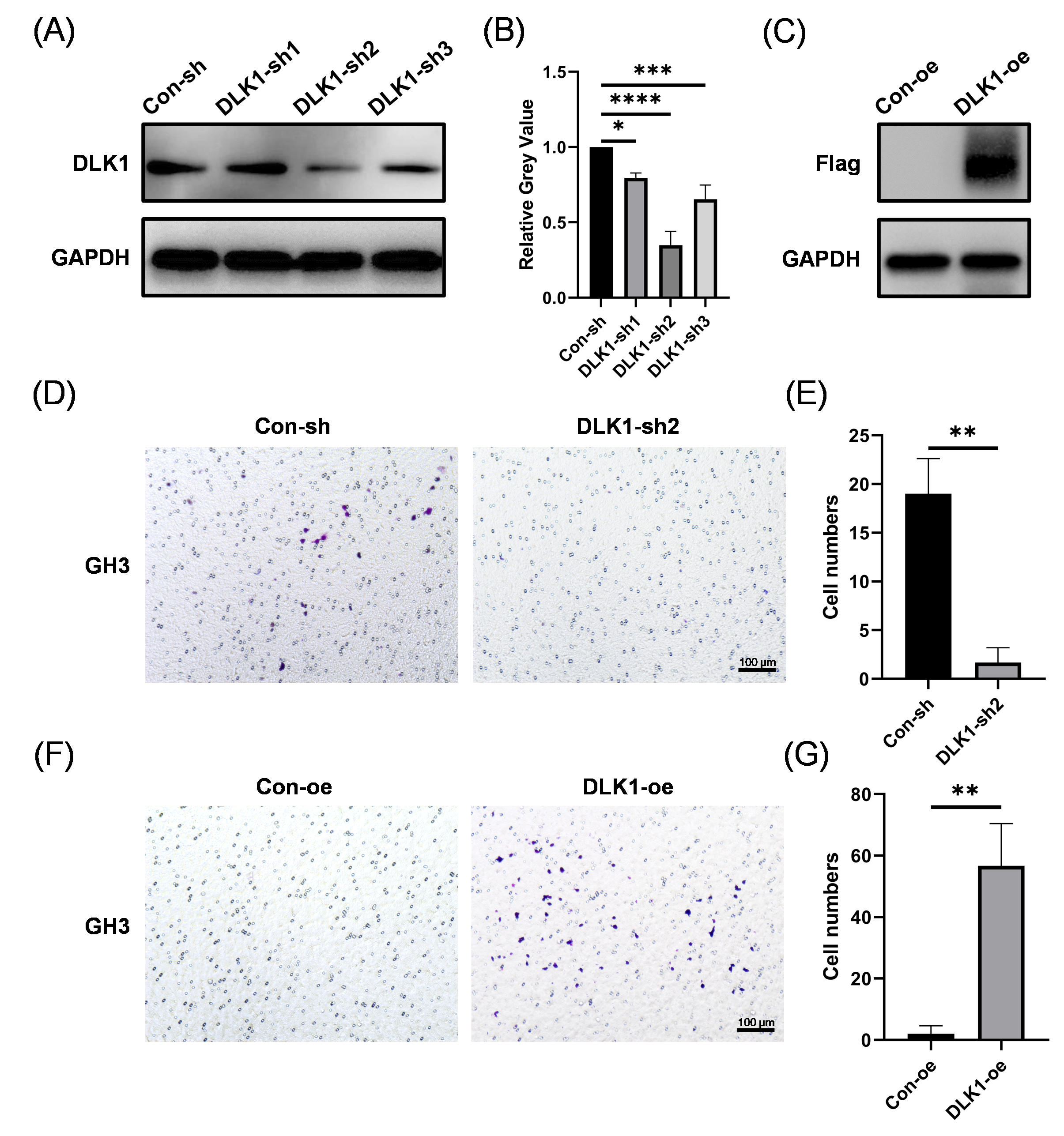

Supplement: Supplementary file 6 — Supporting Information [file CTM2-14-e70090-s004.tif]

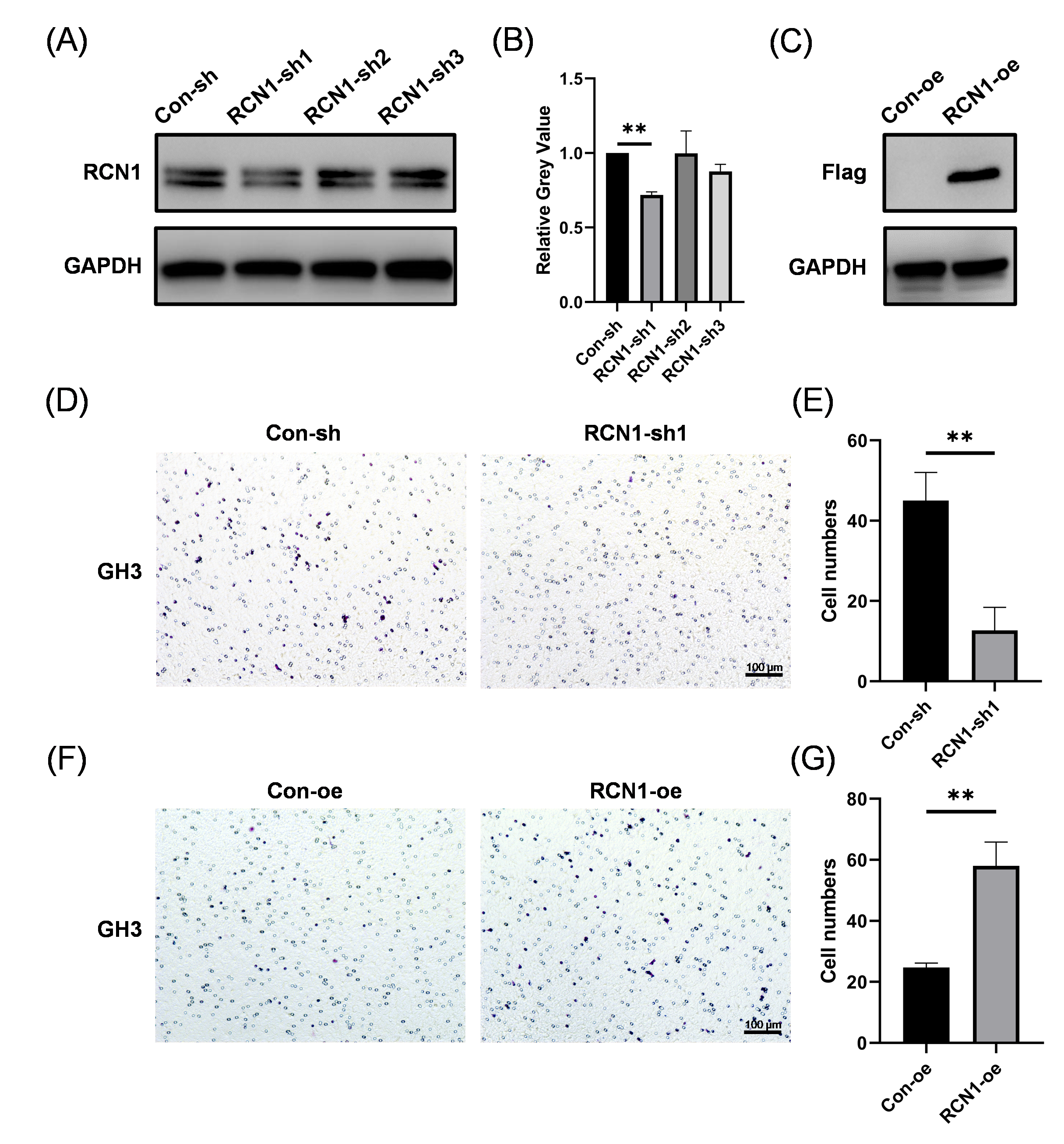

Supplement: Supplementary file 7 — Supporting Information [file CTM2-14-e70090-s007.tif]

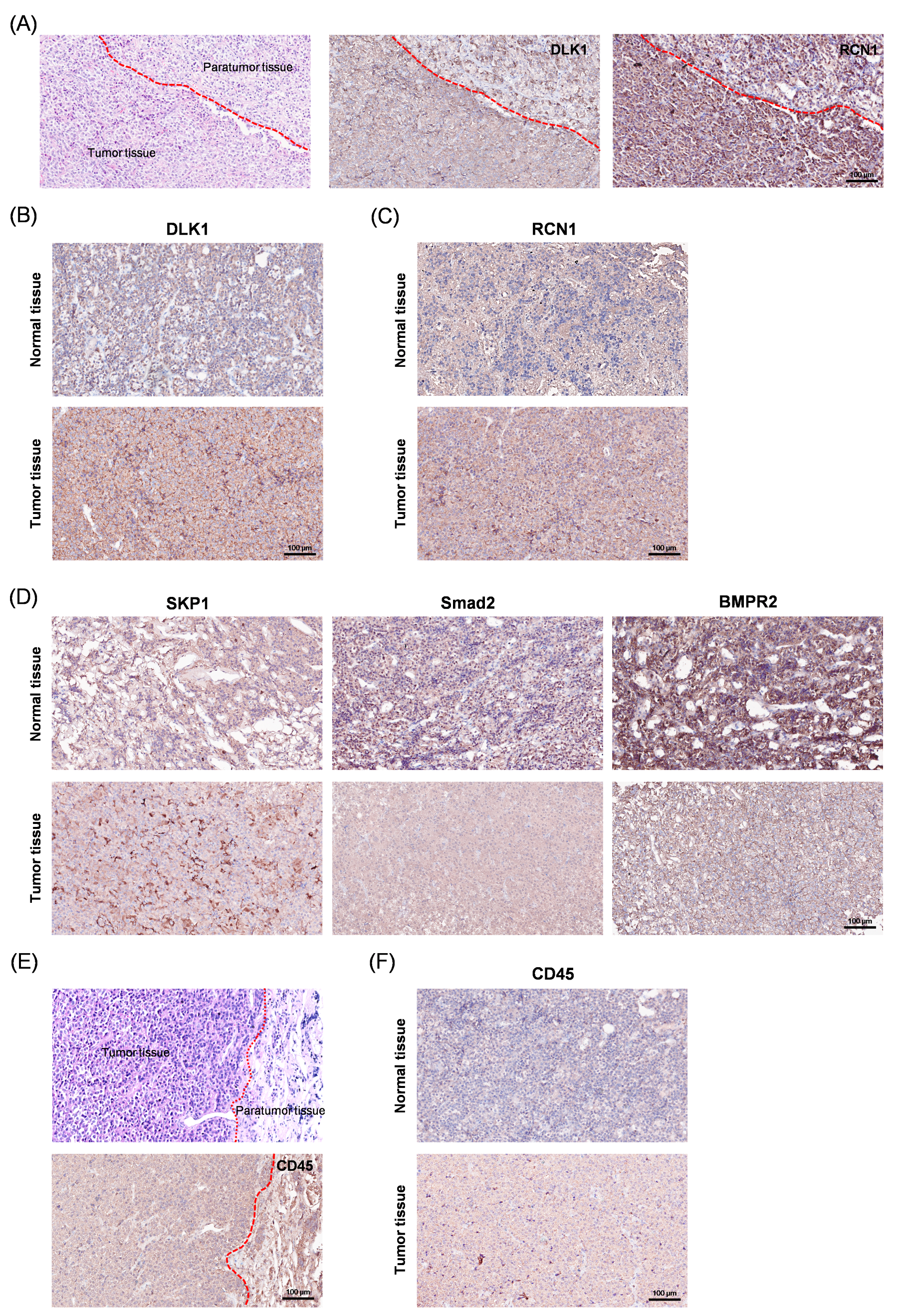

Supplement: Supplementary file 8 — Supporting Information [file CTM2-14-e70090-s012.tif]

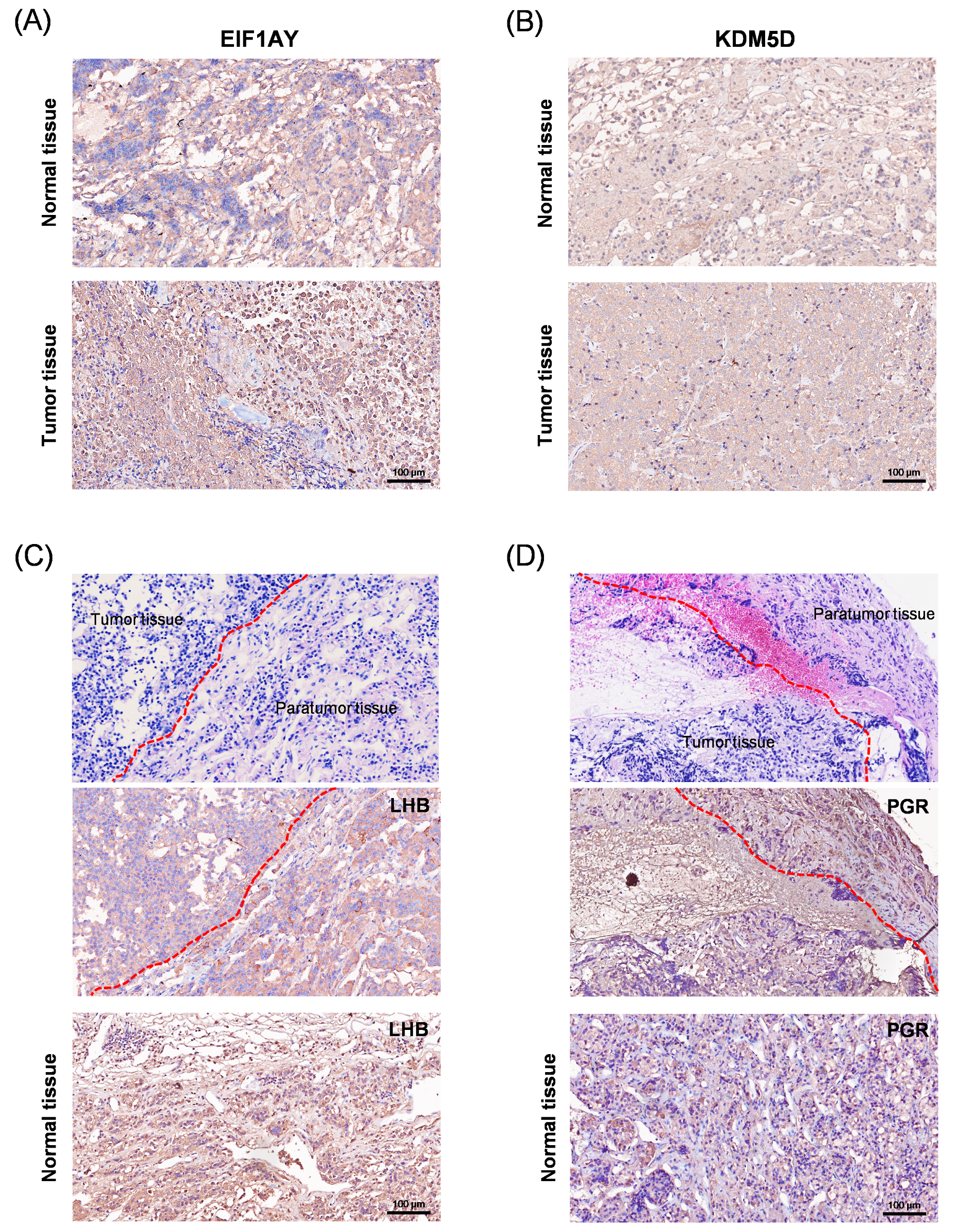

Supplement: Supplementary file 9 — Supporting Information [file CTM2-14-e70090-s002.tif]

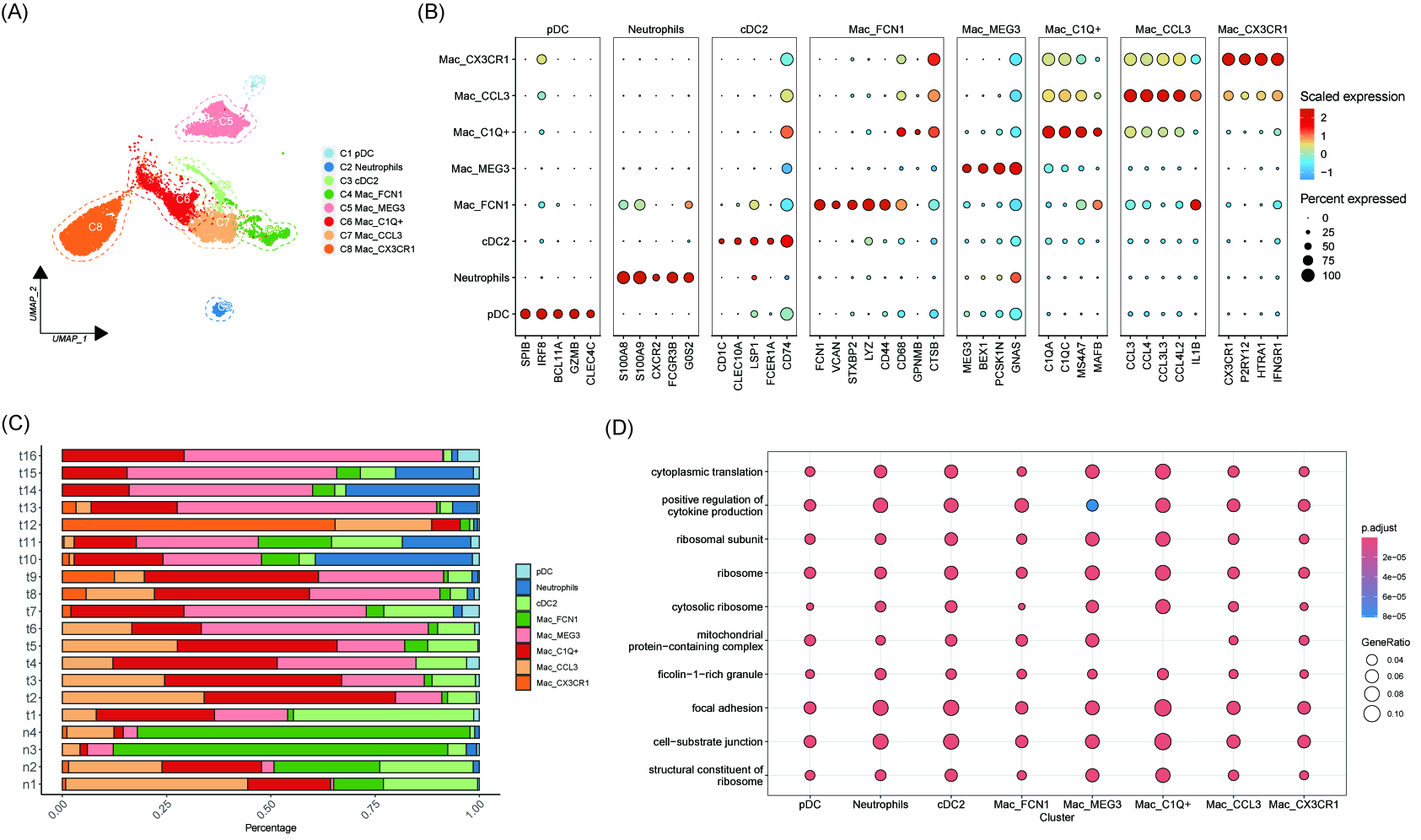

Supplement: Supplementary file 10 — Supporting Information [file CTM2-14-e70090-s003.tif]

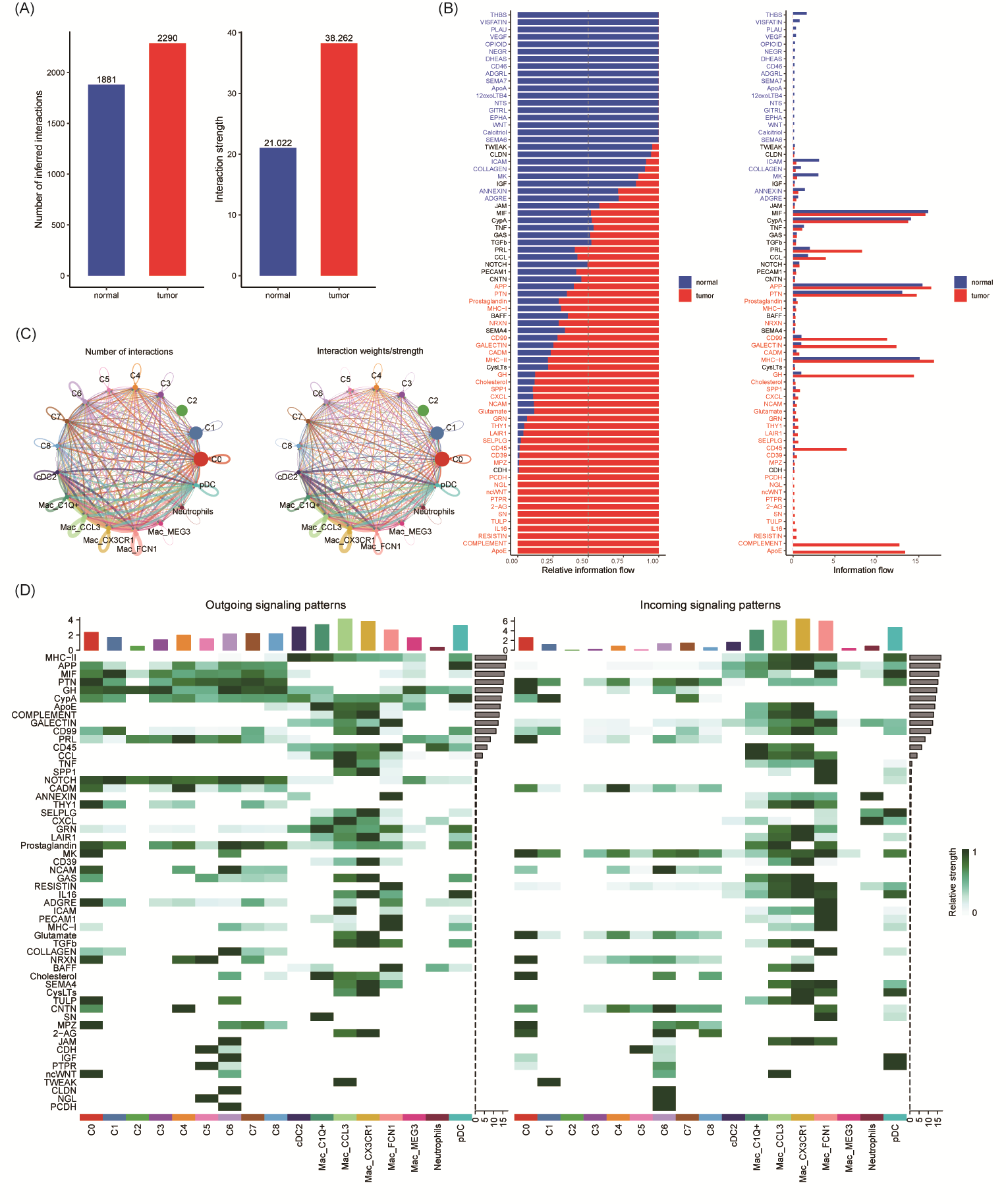

Supplement: Supplementary file 11 — Supporting Information [file CTM2-14-e70090-s009.tif]
