# Supplementary material for: Comprehensive mapping of somatotroph pituitary neuroendocrine tumour heterogeneity using spatial and single‐cell transcriptomics
Source: Clin Transl Med. 2024 Nov 15;14(11):e70090. doi: 10.1002/ctm2.70090 (PMC11567828; doi:10.1002/ctm2.70090)
Supplement: Supplementary file 12 — Supporting Information [file CTM2-14-e70090-s011.docx]

**Supplementary table S1**


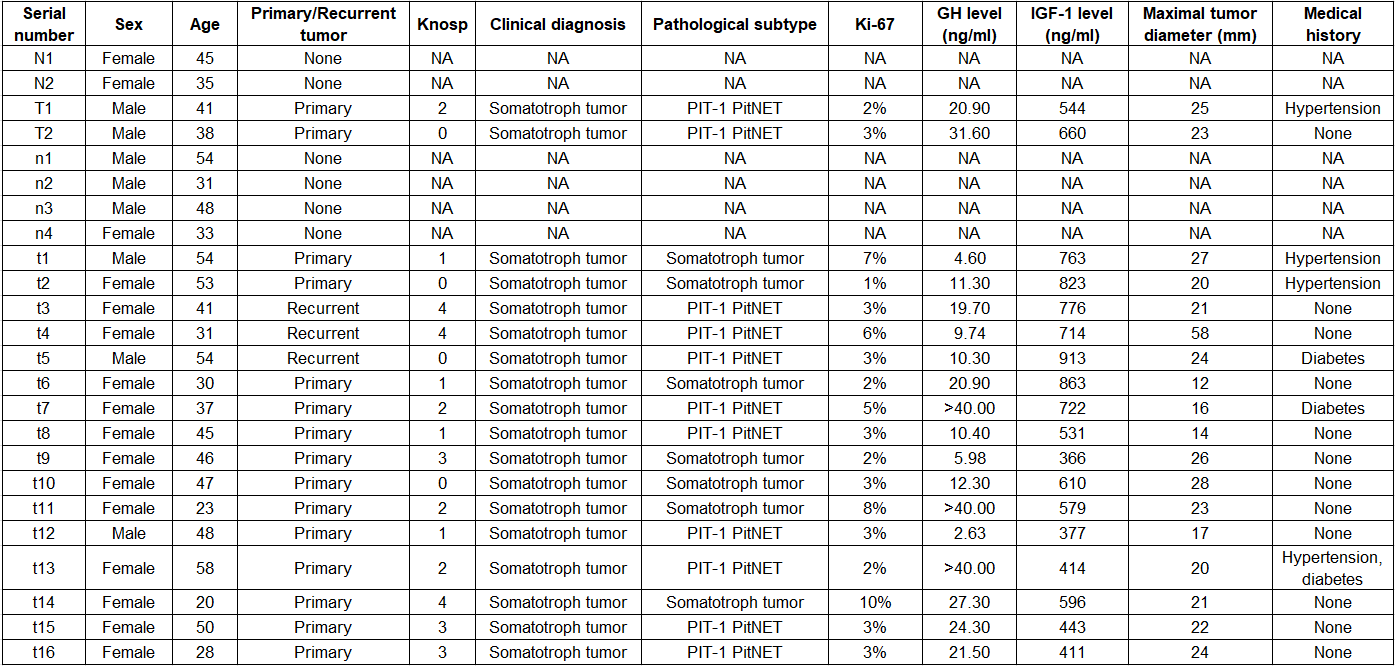


**Supplementary table S2**


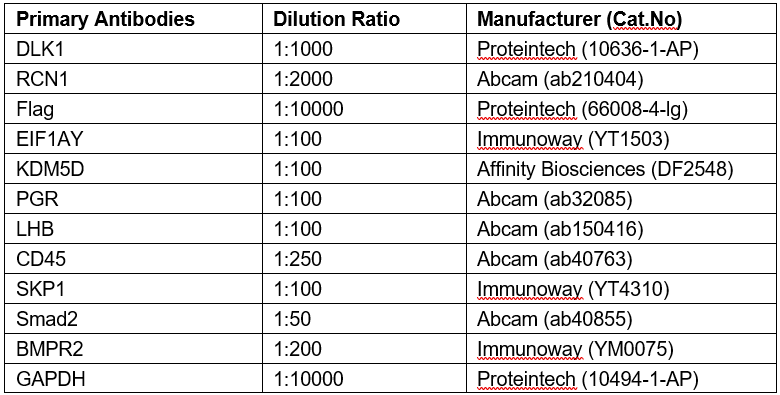


**Supplementary figures**


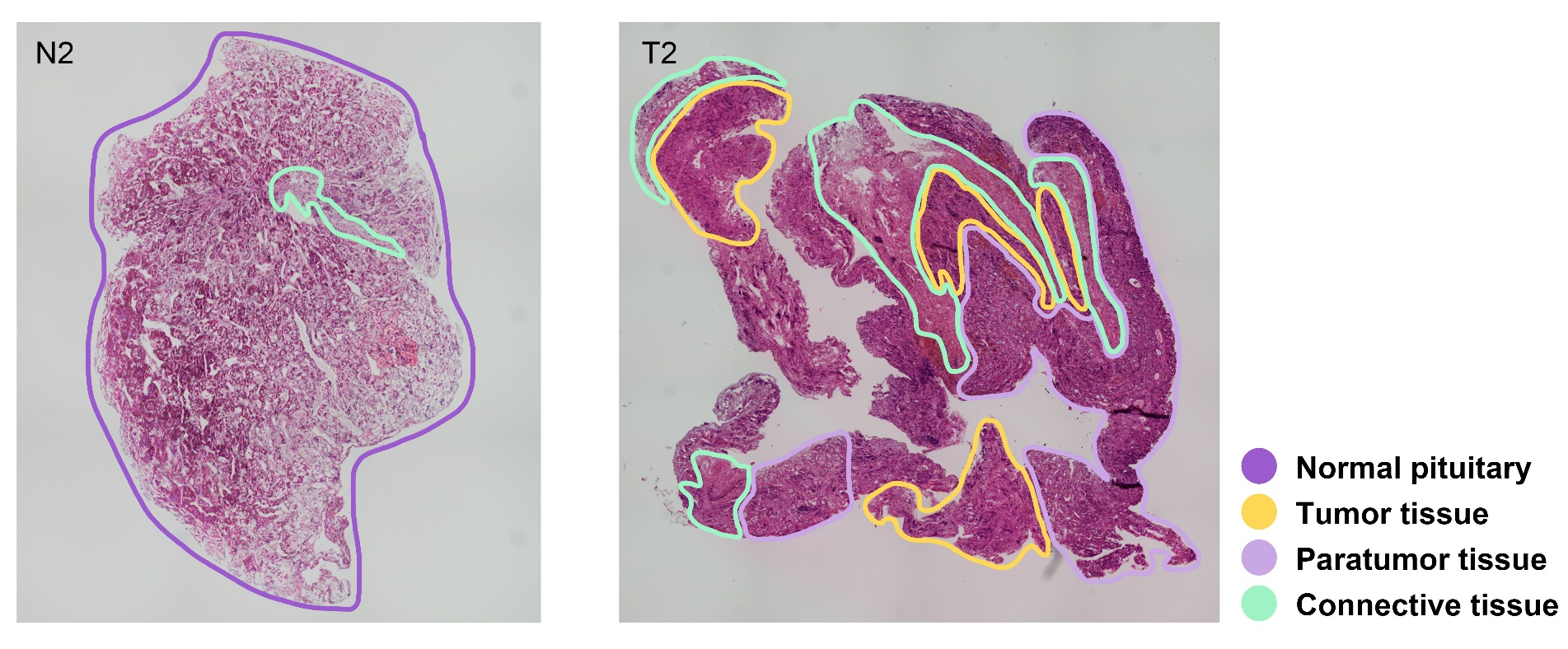


**Figure S1. H&E-stained images of N2 and T2 samples annotated by pathologists.** T2 sample contained tumor area and adjacent paratumor area. Abbreviations: N, normal pituitary; T, tumor tissue.


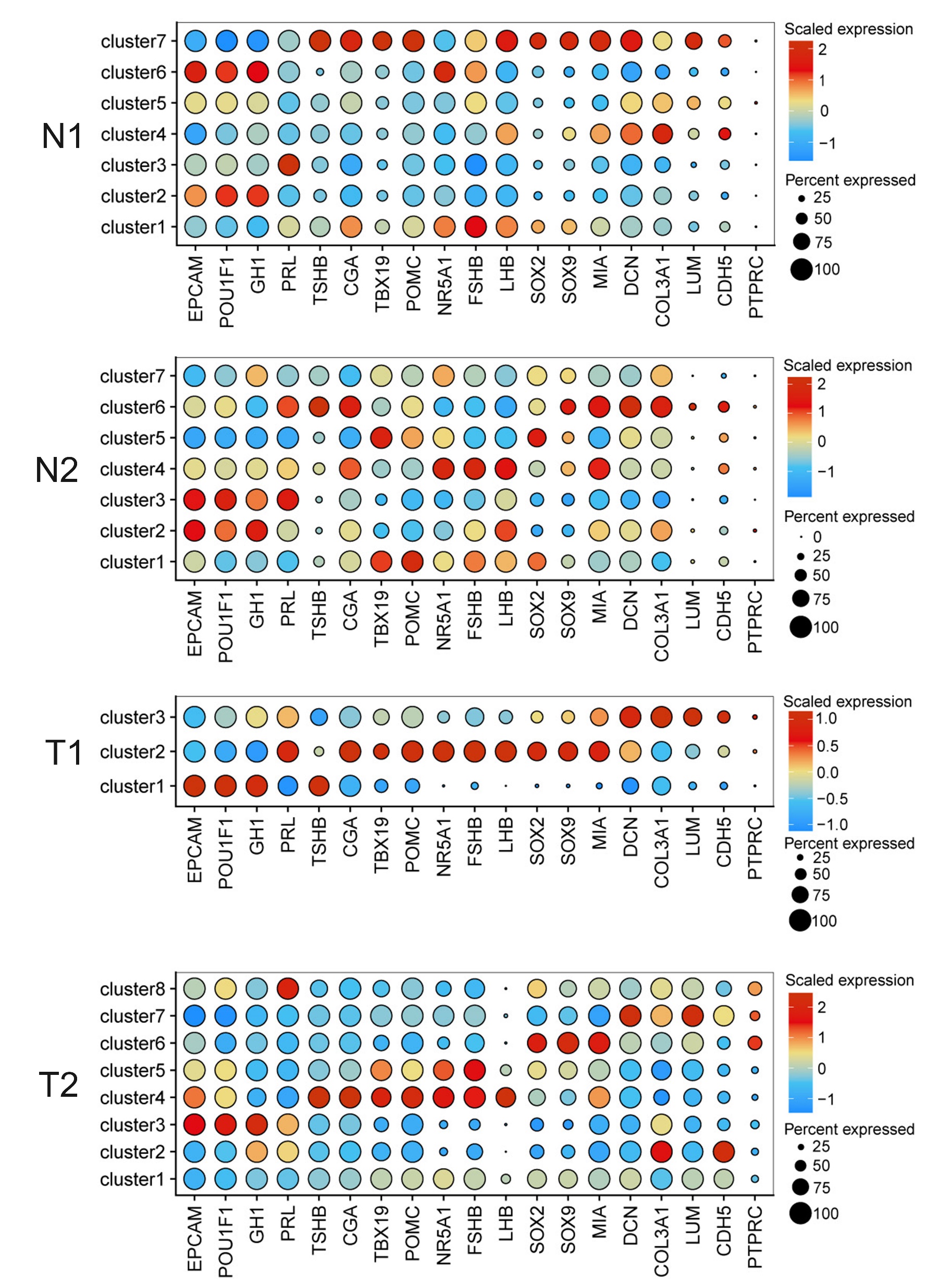


**Figure S2. Expression of known markers in normal pituitary and somatotroph tumor samples.** Bubble charts represented the average expression of marker genes in the indicated cell clusters. The size of the dots represents the percentage of cells in each cluster that express the indicated gene. The shade of color represents the average gene expression level.


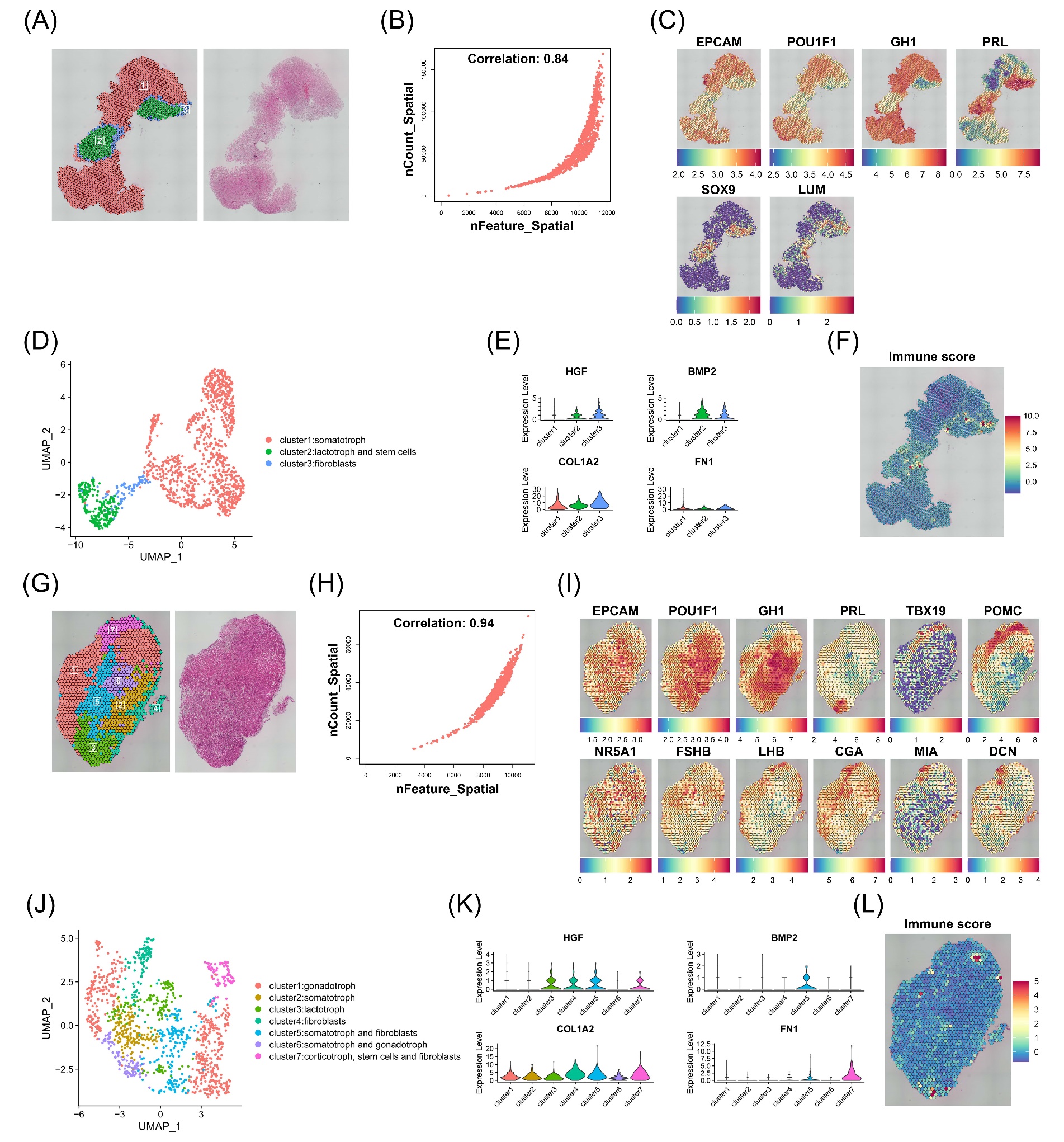


**Figure S3. Cellular identification and characterization of normal pituitary (N1) and somatotroph tumor (T1).** **(A)-(F)** for the tumor tissue (T1). Spatial transcriptomic profile of the T1 sample. Unbiased clustering of ST spots and H&E staining. **(B)** Scatter plot presenting the correlation coefficient between spatial spots and genes was 0.84. **(C)** Spatial plot depicting the marker distribution of somatotroph, lactotroph, stem cells, and fibroblasts. **(D)** UMAP plot showing the cellular landscape of T1. **(E)** Violin plots displaying the expression of genes associated with angiogenesis and tumor invasiveness (HGF, BMP2, COL1A2, and FN1) across the clusters. **(F)** Violin plot showing the immunity score across the clusters. **(G)-(L)** for the normal pituitary (N1), using the same representation as tumor tissue. Abbreviations: N, normal pituitary; T, tumor tissue.­­


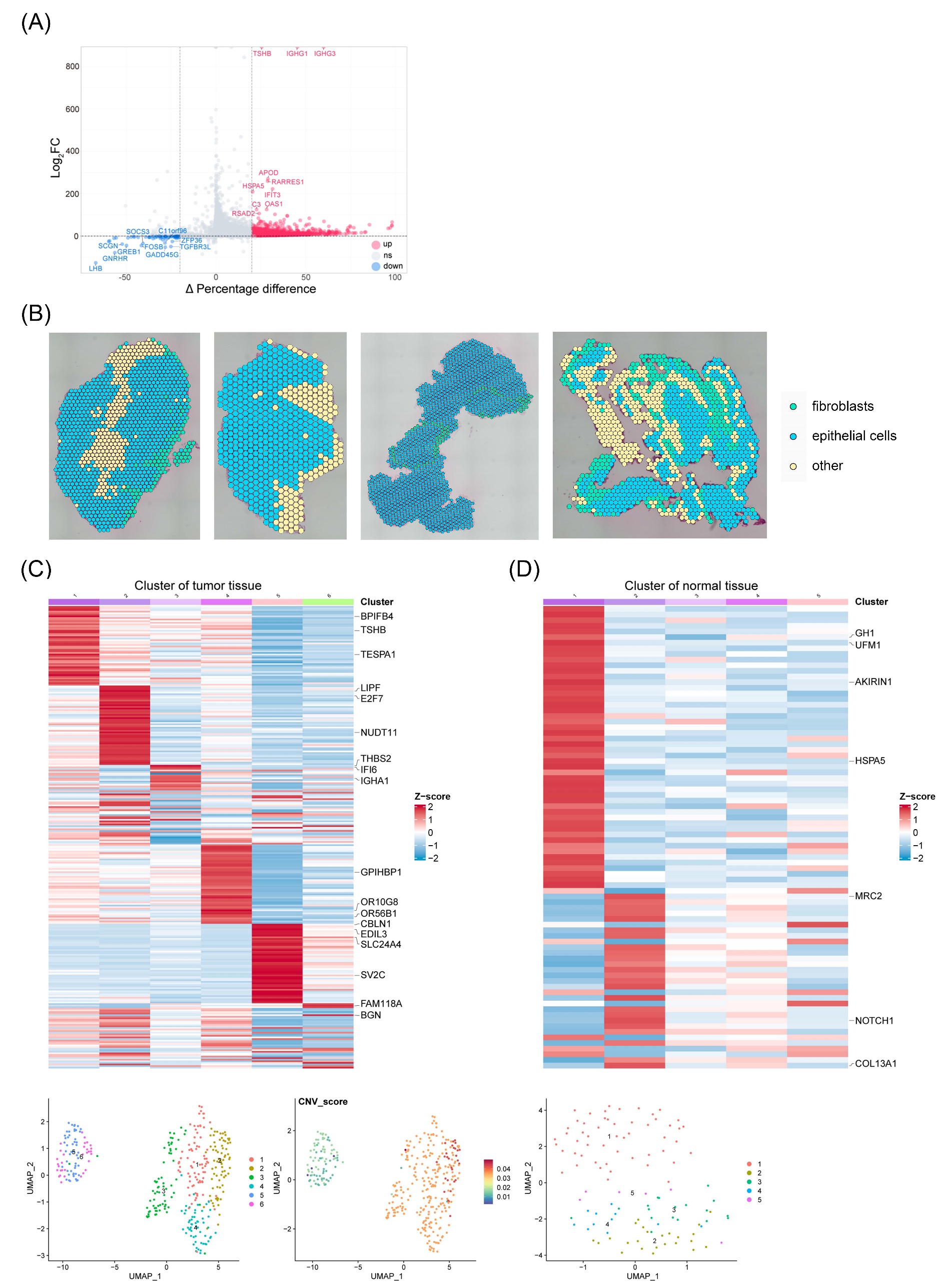


**Figure S4. Differential characteristics in epithelial cells and fibroblasts between normal pituitary and somatotroph tumor.** **(A)** Volcano plot showing DEGs in epithelial cells between the tumor tissue and normal pituitary. **(B)** Fibroblast distributions in four samples. **(C)** Heatmap depicting cluster-specific genes in fibroblasts of tumor tissue and UMAP plot showing unbiased clustering and CNV levels. **(D)** Heatmap showing cluster-specific genes in fibroblasts of normal pituitary and UMAP plot depicting unbiased clustering.


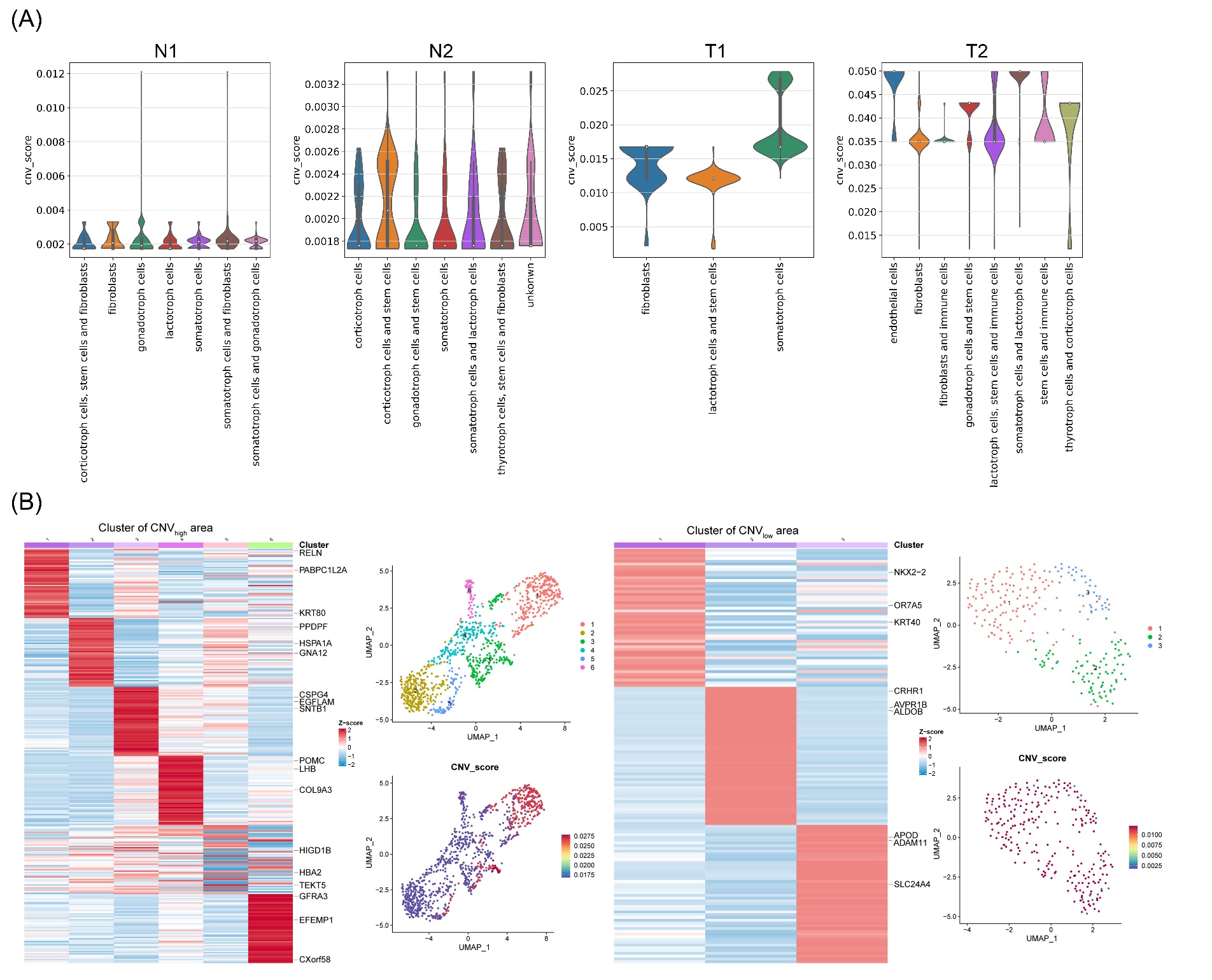


**Figure S5. Inter- and intra-tumor heterogeneity in somatotroph PitNETs.** **(A)** Violin plot showing the CNV scores of clusters in four smaples. **(B)** Heatmap presenting the cluster-specific genes of CNV_high_ or CNV_low_ area in T1 sample. UMAP plot showing spots of CNV_high_ or CNV_low_ area in T1 sample, colored based on unbiased clustering and CNV levels.

­­­

**
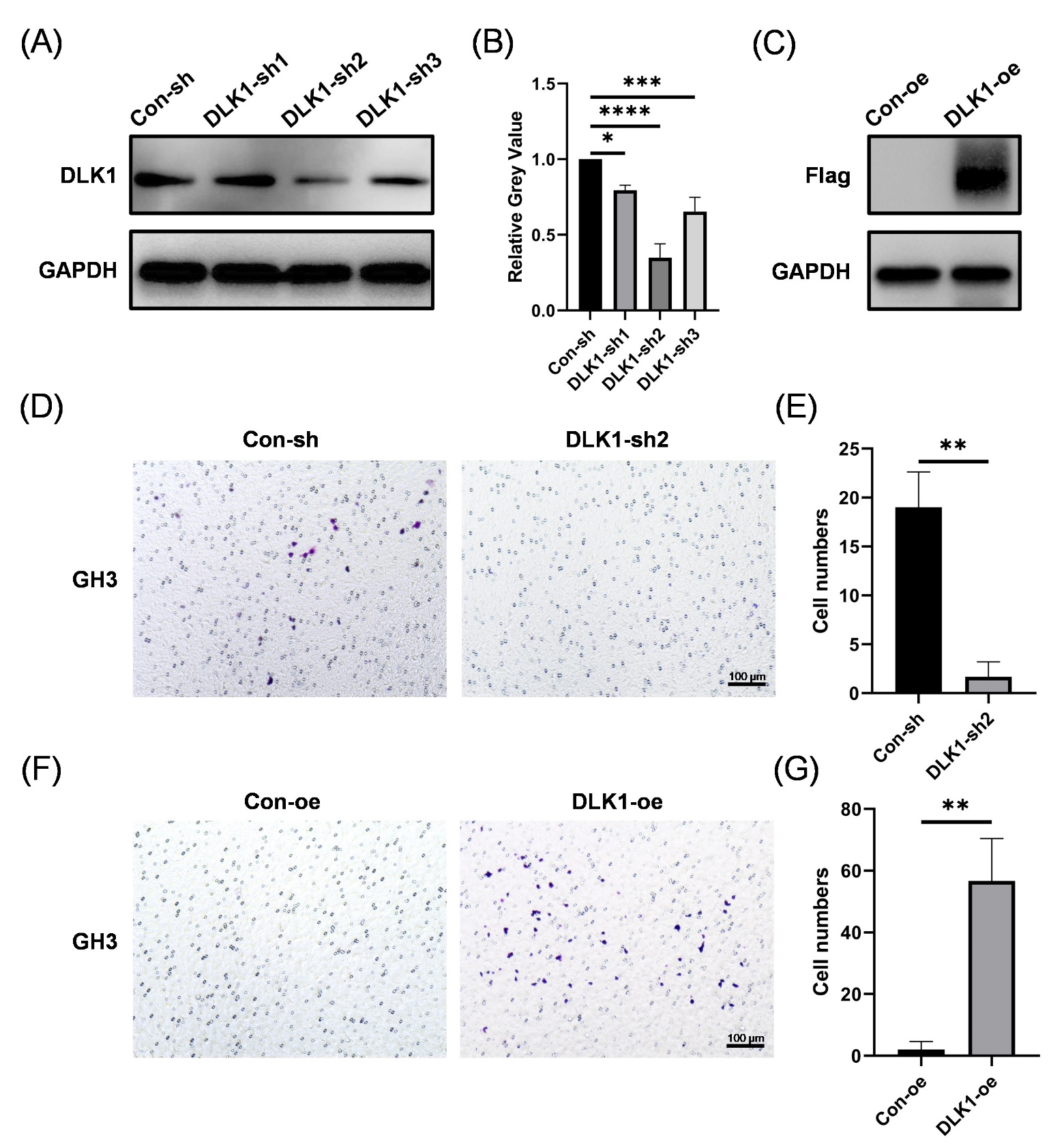
**

**Figure S6 DLK1 promotes cell migration in GH3 cells. (A)** The transfection efficiency of the DLK1 gene was assessed by Western blot compared to the control group. **(B)** Quantification of DLK1 protein expression levels by Western blot. Band intensities were quantified and standardized to GAPDH. **(C)** The transfection efficiency of the DLK1 gene was assessed by Western blot. **(D)** Transwell migration analysis showed that DLK1 knockdown inhibited cell migration (Scale bar: 100 μm). **(F)** Transwell migration analysis showed that DLK1 overexpression promoted cell migration (Scale bar: 100 μm). **(E)** and **(G)** Quantitative analysis of transwell migration assay in groups. * p < 0.05, ** p < 0.01, *** p < 0.001, **** p < 0.0001.

**
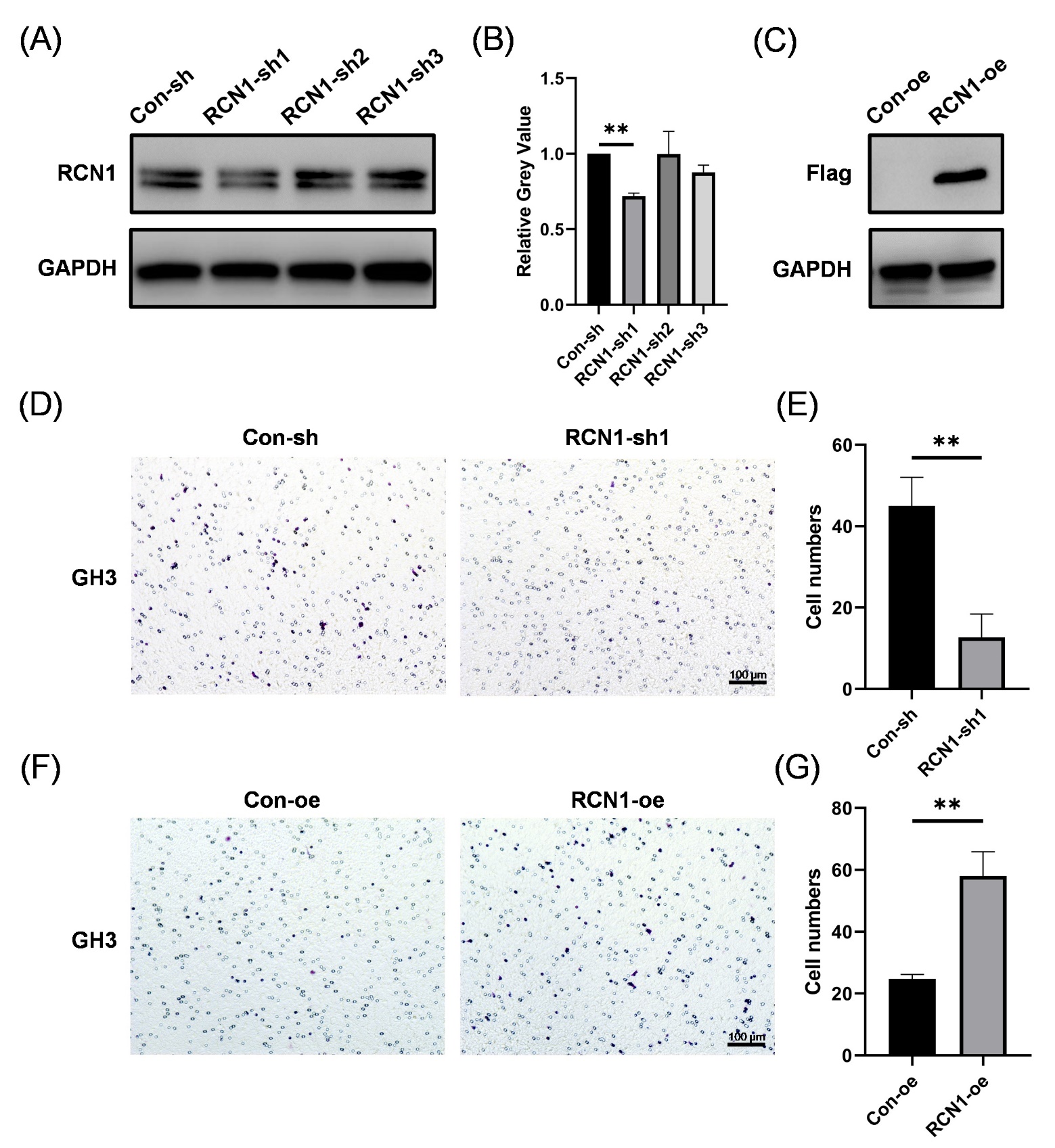
**

**Figure S7 RCN1 promotes cell migration in GH3 cells. (A)** The transfection efficiency of the RCN1 gene was assessed by Western blot compared to the control group. **(B)** Protein expression levels of RCN1 were quantified by Western blot. The band intensities were quantified and standardized to GAPDH. **(C)** Transfection efficiency of RCN1 gene was evaluated by Western blot. **(D)** Transwell migration analysis showed that RCN1 knockdown inhibited cell migration (Scale bar: 100 μm). **(F)** Transwell migration analysis showed that RCN1 overexpression promoted cell migration (Scale bar: 100 μm). **(E)** and **(G)** Quantitative analysis of transwell migration assay in groups. ** p < 0.01.

**
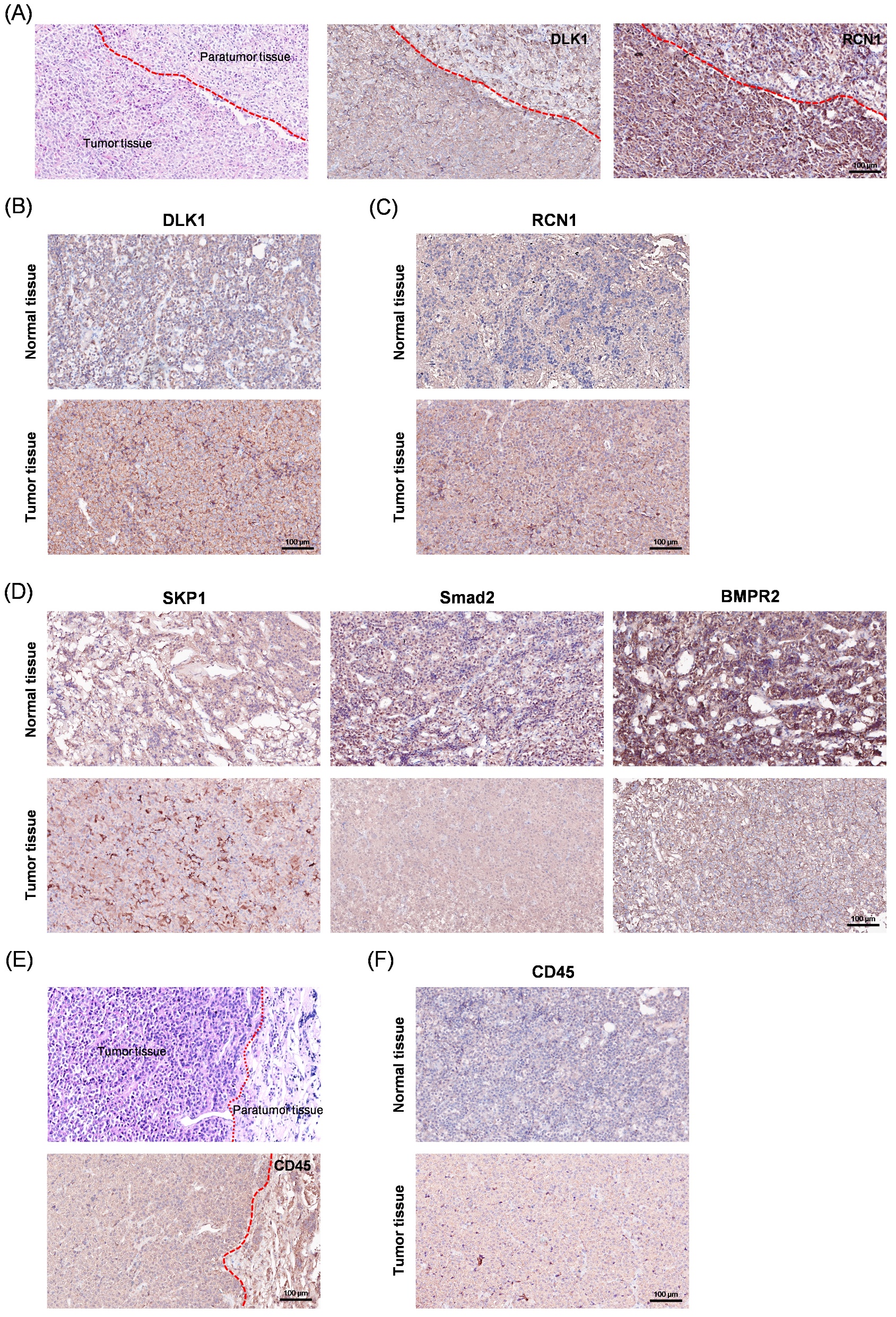
**

**Figure S8 Heterogeneity of differential genes in somatotroph pituitary tumors. (A)** Immunohistochemistry showed the expression of DLK1 and RCN1 in the paratumor tissue and tumor tissue. The red dotted line represents the regional dividing line. **(B)** and **(C)** illustrated the DLK1 and RCN1 expression between normal tissues and tumor tissues. **(D)** Immunohistochemistry assays demonstrated the heterogeneity of TGF-β signaling pathway in pituitary tumors. **(E)** and **(F)** showed expression of the immune cell marker (CD45) in normal, paratumor and tumor regions.


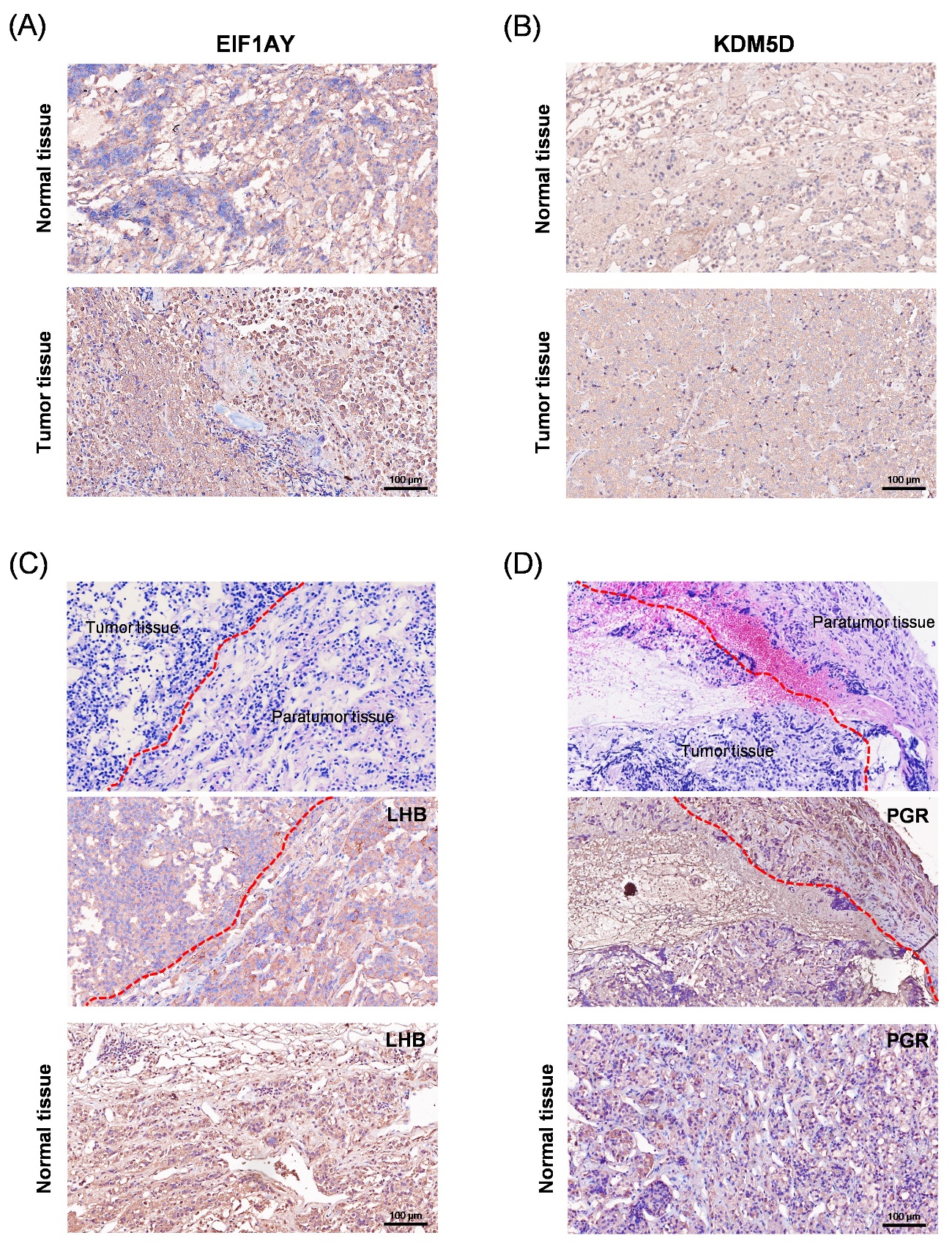


**Figure S9 Expression of differential genes in somatotroph pituitary tumors. (A)** and **(B)** EIF1AY and KDM5D highly expressed in tumor tissues. **(C)** and **(D)** LHB and PGR highly expressed in the normal tissue and paratumor region.


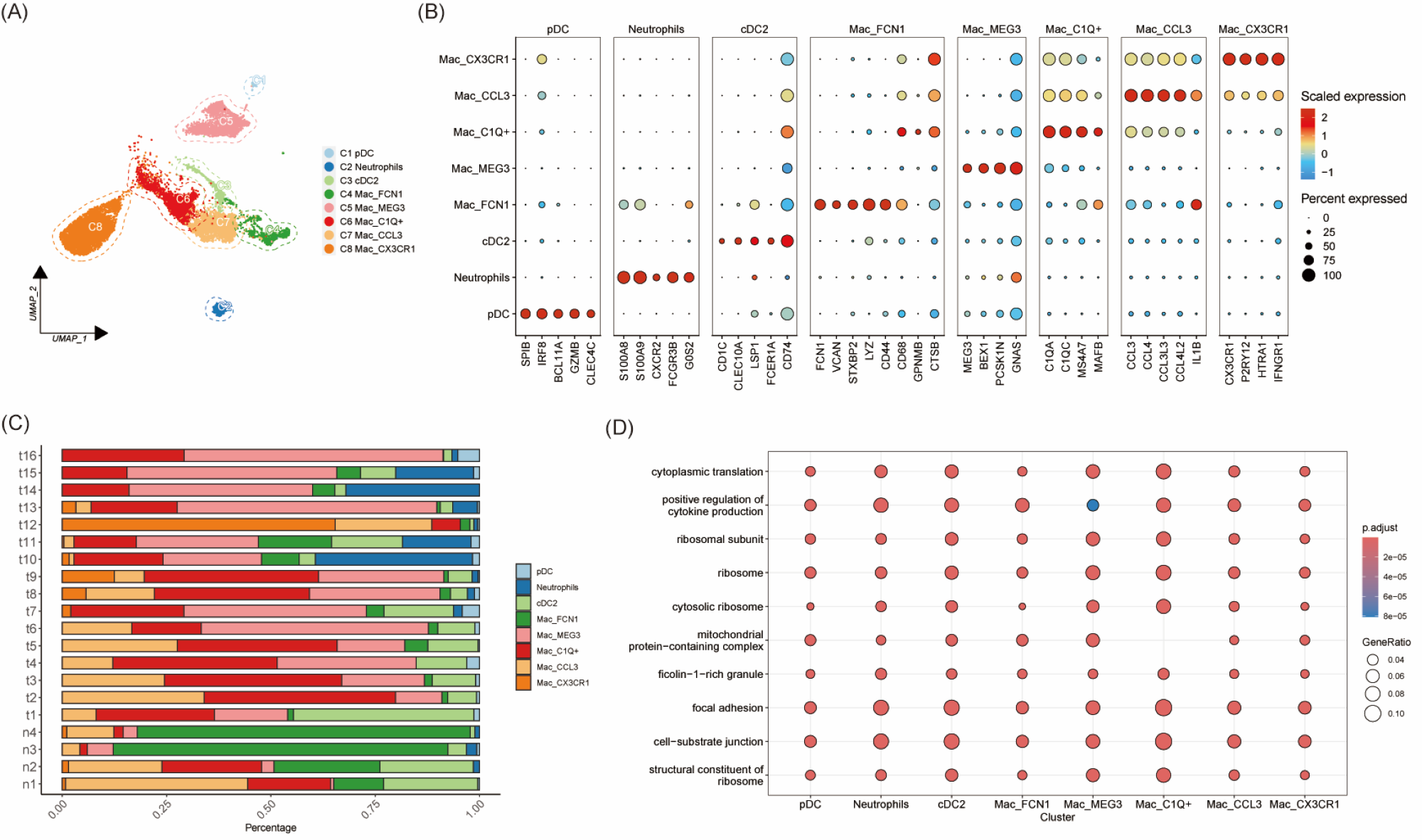


**Figure S10 Single-cell transcriptome characterization of myeloid cells in somatotroph pituitary tumors. (A)** UMAP plots of myeloid cells (macrophage and monocyte) obtained from the normal pituitary and somatotroph tumor tissues. Eight clusters are shown in the UMAP plot. Each cluster is represented using a different color. **(B)** The dot plot showed the average expression of marker genes in the indicated cell cluster. **(C)** The bar plot showed the proportion of eight cell types in different samples. **(D)** The dot plot represented the main function enrichment of DEGs in different clusters.


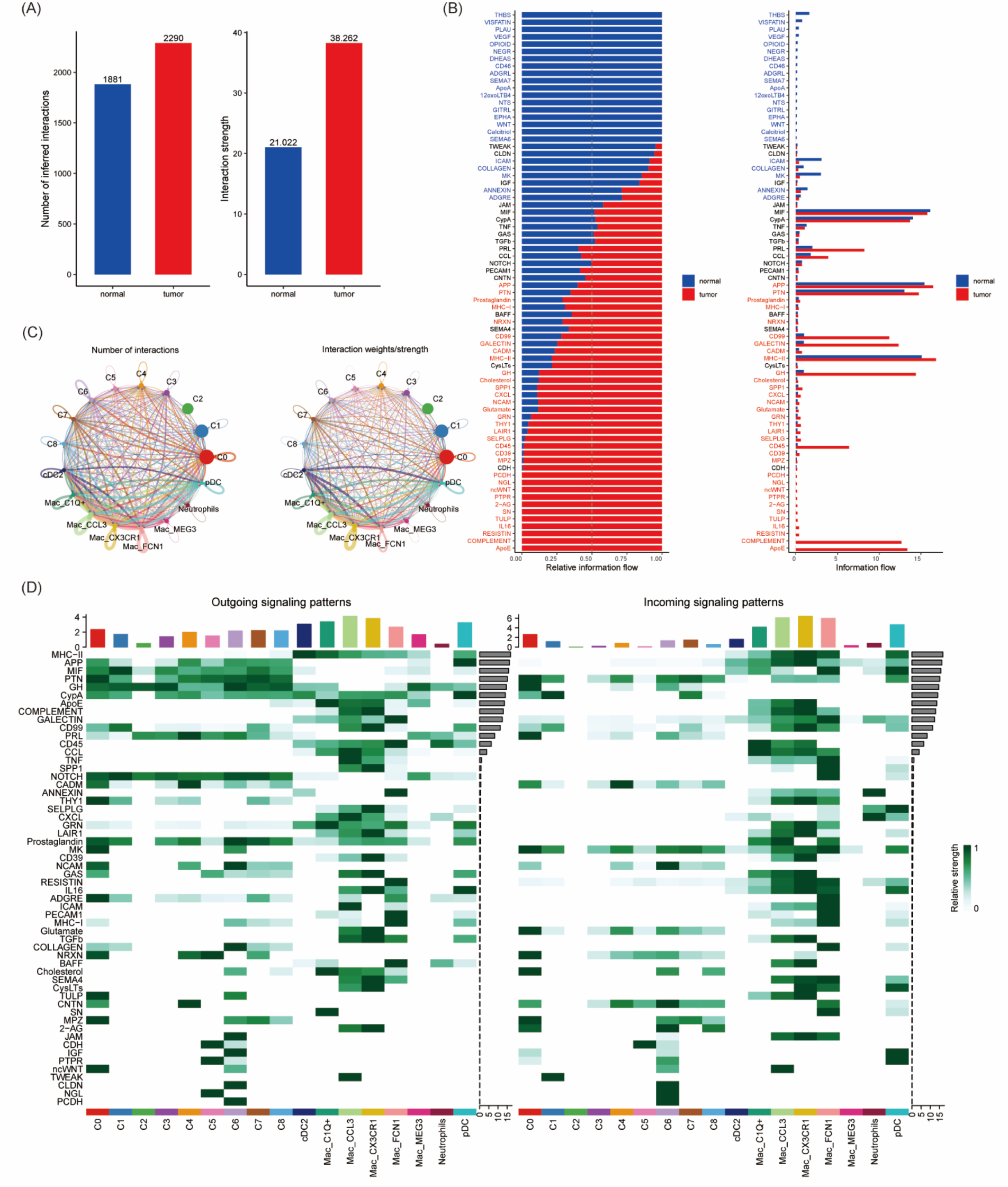


**Figure S11 Cellular communications in normal pituitary and somatotroph tumor samples. (A)** Bar plot showing the number of inferred interaction and strength. **(B)** Bar plot showing the common and specific pathways. *P*-values below 0.05 are considered as significant. Paired Wilcoxon test. **(C)** Circle plot showing the interaction number and strength between tumor epithelial cells and the myeloid cell subtypes. **(D)** Heatmap showing the strength of incoming and outgoing signaling pathways. Top bars show the cumulative signal strength in each cellular population, and right bars show the cumulative strength in each signal.

**Supplementary Methods**

**Plasmid**

The Flag-tagged DLK1 and Flag-tagged RCN1 constructs were subcloned into the plasmid CV702 vector (CMV enhancer-MCS-3FLAG-SV40-Puromycin), separately. The DLK1 (Rat, NM_053744.2) and RCN1 (Rat, NM_001108586.1) overexpression vectors were purchased from Genechem (Shanghai, China).

**Transfection and RNA interference**

A total of 1x10^6^ GH3 cells were seeded in each well of a six-well plate. Following a 24-hour incubation period in a humidified incubator, GH3 cells were transfected with the short hairpin RNA (shRNA) or plasmid using lipofectamine 3000 (Lipo3000, Thermo Fisher, USA), in accordance with the product specifications. The specific shRNAs for DLK1 and RCN1 were purchased from Genechem (Shanghai, China). The sequences of the DLK1 and RCN1 shRNAs are as follows:

DLK1-Rat-1, 5’ GAAAGGACTGTCAGCACAAGGCTCGAGCCTTGTGCTGACAGTCCTTTC 3’

5’ GAAAGGACTGTCAGCACAAGGCTCGAGCCTTGTGCTGACAGTCCTTTC 3’

DLK1-Rat-2, 5’ GCAACTTCTGTGAGATCGTGA CTCGAG TCACGATCTCACAGAAGTTGC 3’

5’ GCAACTTCTGTGAGATCGTGACTCGAGTCACGATCTCACAGAAGTTGC 3’

DLK1-Rat-3, 5’ GGACGGGAAATTCTGCGAAACTCGAGATTTCGCAGAATTTCCCGTCC 3’

5’ GGACGGGAAATTCTGCGAAATCTCGAGATTTCGCAGAATTTCCCGTCC 3’

RCN1-Rat-1, 5’ GGATTCCAAGACCTTCGATCACTCGAGTGATCGAAGGTCTTGGAATCC 3’

5’ GGATTCCAAGACCTTCGATCACTCGAGTGATCGAAGGTCTTGGAATCC 3’

RCN1-Rat-2, 5’ GCTAACCAAGGAGGAGATTCTCTCGAGAGAATCTCCTCCTTGGTTAGC 3’

5’ GCTAACCAAGGAGGAGATTCTCTCGAGAGAATCTCCTCCTTGGTTAGC 3’

RCN1-Rat-3, 5’ GAACGGGAGCAGTTCAATGATCTCGAGATCATTGAACTGCTCCCGTTC 3’

5’ GAACGGGAGCAGTTCAATGATCTCGAGATCATTGAACTGCTCCCGTTC 3’

**Western blotting**

The GH3 cells were lysed with RIPA buffer (NCM Biotech, China). The protein concentration was determined using the BCA Protein Assay Kit (Thermo Fisher, USA). The proteins were separated by SDS-PAGE and subsequently transferred to PVDF membranes (Millipore, USA). The membrane was blocked and incubated overnight at 4 °C with the diluted primary antibodies (Table S2), followed by incubation with the secondary antibody. GAPDH was used as internal control, and the grey value was calculated using the ImageJ software.

**Transwell migration assay**

GH3 cells (1x10^6^ per well) were seeded in six-well plates. After 24 hours of incubation in the humidified incubator, the GH3 cells were transfected with shRNA or plasmid using Lipofectamine 3000 (Lipo3000, Thermo Fisher, USA) in accordance with the product specification. Subsequently, GH3 cells (5 × 10^5^ per well) were seeded in an upper chamber of a 24-well culture plate (Corning, USA) with 8-μm pores. Over a 40-hour period incubation at 37°C and 5% CO_2_, GH3 cells were allowed to migrate through the pores in the transwell membrane. Then, cells on the lower surface of the membrane were fixed in 4% paraformaldehyde for 15 minutes, stained with crystal violet for a further 10 minutes, and washed with PBS. The migrated GH3 cells were observed and counted using ImageJ software.

**Hematoxylin-eosin staining and immunohistochemistry**

Tumor samples were fixed in 4% paraformaldehyde and subsequently embedded in paraffin after collection. The hematoxylin-eosin (H&E) staining procedure was performed as previously described. Following deparaffinization and antigen retrieval, the 4-μm slides were rinsed in PBS and incubated overnight at 4 °C with the primary antibody (Table S2). Following a one-hour incubation period with the secondary antibody and subsequent staining, the slides were dehydrated and sealed with neutral resin. The images were obtained using the Leica Aperio AT2 and the Leica DM IRB system.
